# Snail1 expression in endothelial cells controls growth, angiogenesis and differentiation of breast tumors

**DOI:** 10.7150/thno.61881

**Published:** 2021-06-16

**Authors:** David Cabrerizo-Granados, Raúl Peña, Laura Palacios, Laura Carrillo-Bosch, Josep Lloreta-Trull, Laura Comerma, Mar Iglesias, Antonio García de Herreros

**Affiliations:** 1 Programa de Recerca en Càncer, Institut Hospital del Mar d'Investigacions Mèdiques (IMIM), Unidad Asociada al CSIC, Barcelona, Spain.; 2 Departament de Ciències Experimentals i de la Salut, Universitat Pompeu Fabra, Barcelona, Spain.; 3 Servei d'Anatomia Patològica, Hospital del Mar, Barcelona, Spain.

## Abstract

Snail1 is a transcriptional factor required for epithelial to mesenchymal transition and activation of cancer-associated fibroblasts (CAF). Apart from that, tumor endothelial cells also express Snail1. Here, we have unraveled the role of Snail1 in this tissue in a tumorigenic context.

**Methods**: We generated transgenic mice with an endothelial-specific and inducible Snail1 depletion. This murine line was crossed with MMTV-PyMT mice that develop mammary gland tumors and the consequence of Snail1 depletion in the endothelium were investigated. We also interfere Snail1 expression in cultured endothelial cells.

**Results**: Specific Snail1 depletion in the endothelium of adult mice does not promote an overt phenotype; however, it delays the formation of mammary gland tumors in MMTV-PyMT mice. These effects are associated to the inability of Snail1-deficient endothelial cells to undergo angiogenesis and to enhance CAF activation in a paracrine manner. Moreover, tumors generated in mice with endothelium-specific Snail1 depletion are less advanced and show a papillary phenotype. Similar changes on onset and tumor morphology are observed by pretreatment of MMTV-PyMT mice with the angiogenic inhibitor Bevacizumab. Human breast papillary carcinomas exhibit a lower angiogenesis and present lower staining of Snail1, both in endothelial and stromal cells, compared with other breast neoplasms. Furthermore, human breast tumors datasets show a strong correlation between Snail1 expression and high angiogenesis.

**Conclusion**: These findings show a novel role for Snail1 in endothelial cell activation and demonstrate that these cells impact not only on angiogenesis, but also on tumor onset and phenotype.

## Introduction

Since it was characterized as a repressor of E-Cadherin gene (CDH1) expression and an inducer of epithelial-mesenchymal transition (EMT) 1, 2, the transcriptional factor Snail1 has attracted the attention from many cancer cell biologists. Snail1 ectopic expression in epithelial cells promotes an EMT and increases several features associated to this conversion; cells become more invasive and resistant to apoptotic insults, reprogram their metabolism, acquire characteristics of stem cells and secrete cytokines protecting them from the immune attack 3, 4. Moreover, Snail1 is rapidly induced by cytokines and other stimuli promoting EMT and precedes the expression of other mesenchymal markers 5-7.

Snail1 is also expressed by cancer-activated fibroblasts (CAF). In these cells, Snail1 is induced by cytokines promoting fibroblast activation such as TGFβ and PDGFb and is required for the expression of CAF-specific proteins 8, 9. In accordance with this dual role in EMT and fibroblast activation, Snail1 depletion in adult transgenic mice retards breast tumor development and prevents metastasis 10, 11.

Expression of Snail1 has been assessed in multiple tumors and related with a poor prognosis 12. In most neoplasms, Snail1 is predominantly detected in CAF although some epithelial cells placed in the area of invasion do also express it 13. When analyzing several types of human tumors, we have detected Snail1 expression in a subset of endothelial cells. Actually, several groups have previously reported that Snail1 is expressed in vascular cells during development and that Snail1 depletion alters vascular development in embryos 14, 15 and the proper development of retina 16, 17. These results suggest that Snail1 might control tumor angiogenesis; thus, the activation of endothelial cells by tumor-derived factors creating an aberrant vascular network that grows and infiltrates tumors to supply them oxygen and nutrients 18. In this report, we have analyzed the role of Snail1 in the tumor endothelial cells using the well stablished breast cancer model of MMTV-PyMT 19. We describe that Snail1 depletion in endothelium, besides controlling angiogenesis, severely affects tumor onset, morphology and malignancy.

## Materials and Methods

### Cell Culture

Authenticated human microvascular endothelial cells (HMEC-1) were a kind gift of Dr. MI Díaz-Ricart (Hospital Clínic, Barcelona, Spain). Wild type Mouse Embryonic Fibroblasts (MEF) were previously established in our laboratory 9, 11. HMEC-1 were grown in MCDB131 medium supplemented with EGF (10 ng/mL), Hydrocortisone (1 µg/mL), Glutamine (10 mM) and fetal bovine serum (FBS, 10%); MEF, in Dulbecco's modified Eagle's medium (DMEM) supplemented with FBS (10%). All cells were cultured at 37 ºC in a humidified incubator (HeracellTM 150) with 5% CO_2_ and were periodically tested to verify that they remained mycoplasma-free.

Snail1 interference in HMEC-1 was accomplished using a siRNA against Snail1 (Dharmacon, SNAI1-targeting ON-TARGETplus siRNA pool, cat. No L-010847-01-0005); a siRNA control (Dharmacon, ON-TARGETplus Control siRNA, cat. No. D-001810-02-50) was also transfected also using DharmaFECT transfection agent (Dharmacon). In some experiments, cells were treated with VEGFA (Preprotech, 10-20), FGF2 (Merck, GF003), and the inhibitors of TGFβ receptor SB505125 (SB, S6496, Sigma) or PDGF receptor Crenolanib (CRN, S2730, Selleck).

### Immunofluorescence

Cultured cells were fixed with p-formaldehyde (4%) in PBS during 10 min at 4 ºC and permeabilized during 10 min with Triton X-100 (1%) in PBS at 4 ºC. Afterwards, cells were blocked with PBS plus bovine seroalbumin (BSA) (2%) during 1 h at RT. Primary antibodies (Table S1) and the corresponding Alexa-conjugated secondary antibodies (Life Technologies) were diluted in blocking solution and incubated during 1 h at RT. DAPI (Sigma) was used for counterstain nuclei and samples were mounted with Fluoromount-G. Leica SP5 confocal microscope was used for imaging at Advanced Light Microscopy Unit facility of the Parc de Recerca Biomèdica de Barcelona/Centre de Regulació Genòmica (PRBB/CRG).

### Human samples collection and analysis

Human tumor samples were obtained from Parc de Salut MAR Biobank (MARBiobanc), Barcelona. Their study was approved by the Ethical Committee for Clinical Research from PRBB.

The co-expression data of mRNA and protein and survival curves of the two groups mimicking the PyMT-VE-Cadh^Snail1CT^ and PyMT-VE-Cadh^Snail1KO^ mice were obtained from TCGA Breast Invasive Carcinoma (Firehose Legacy) data in the cBioPortal public database (http://www.cbioportal.org/) on May, 2020 20, 21. In this study we assessed the expression of *PECAM1* (CD31), *VIM*, *SNAI1*, *KRT5*, *KRT14*, *TP63* and *PTPRC* (CD45).

### Immunohistochemistry

Samples were fixed with p-formaldehyde (4%) at room temperature. When indicated, consecutive sections of tumors were used. Fixed samples were dehydrated and paraffin-embedded according standard procedures. Sections (2.5 µm) were prepared for immunohistochemical analysis and stained with hematoxylin and eosin or Masson's trichrome stain for histological or Collagen content evaluation, respectively. After standard deparaffination and rehydration of the samples, antigen unmasking was carried immersing the sections in Tris EDTA buffer pH 9 or citrate buffer pH 6 and boiling for 15 min. Samples were blocked during 2 h in Tris-buffered saline (TBS) plus FBS (1%) and BSA (1%), and later incubated with primary antibodies overnight (Table S1). Signal was amplified with EnVision+ System HRP Labelled Polymer (anti mouse or anti rabbit, DAKO) and visualized with the DAB kit (DAKO).

For double immunohistochemistry, after boiling in Citrate buffer pH 6 boiling for unmasking, tumor samples were blocked with BSA (2%) and incubated with anti Snail1 antibody overnight. Signal was amplified with EnVision+ System HRP Labelled Polymer (anti-rabbit, DAKO) by overnight incubation and visualized with the DAB kit (DAKO). Afterwards, slides were extensively washed, blocked with BSA (2%) and incubated with anti Von Willebrand Factor VIII antibody overnight. Signal was amplified overnight with anti-rabbit Alkaline Phosphatase conjugated (DAKO), visualized with Permanent-Red kit (NeoBiotech) and counterstained with hematoxylin. Slides were imaged either with Aperio ScanScope (Leica) at the Anatomy Department, Hospital del Mar, or with Olympus BX61 microscope at IMIM Microscopy Unit.

For the analysis of tumor vasculature, a minimum of five randomized areas of each CD31 stained tumor slide (three animals per group) were captured using an Olympus BX61 microscope at 10x magnification. CD31 stained area was measured using ImageJ software and microvessel density was quantified as the percentage of CD31+ area per image area. Open vessels of the images were identified and manually delimited using ImageJ software. Lumen size of the vessel was obtained by the software and the mean of individual vessel lumen size was represented in each condition. Other methods used for the quantification of the immunohistological analyses are presented in Supplemental Information.

### Animals

Animals were housed in a specific pathogen-free area and fed *ad libitum* at the PRBB Animal Facility. All the animal procedures were previously approved by the Animal Research Ethical Committee from the PRBB and by the Generalitat de Catalunya. The generation of the murine line bearing a Snail1 floxed allele (Snail1 flox) and a Snail1-wild-type (Snail1) or Snail1-deleted (Snail1 del) allele has been previously described 9. This line was crossed in a C57/Bl6 background with Cdh5-CreERT2 mice line 22 to generate an endothelium-specific Snail1 depletion. These mice were referred as VE-Cadh^Snail1CT^ and VE-Cadh^Snail1KO^. Depletion of Snail1 in those mice was performed by five intraperitoneal daily doses of Tamoxifen (0.2 mg/g) in 6-weeks old mice 20.

VE-Cadh^Snail1CT/KO^ mice were mated with mice holding the Polyoma Middle T antigen (PyMT) under the control of MMTV promoter (MMTV-PyMT mice). These mice develop spontaneously Luminal B mammary gland tumors 19. Tamoxifen induction was performed as above and an additional dose was administrated every three weeks until animals were euthanized. When indicated, mice were treated with Bevacizumab (Avastin, Roche) (50 mg/kg) diluted in PBS twice per week, starting when they were six weeks old. Mice were palpated twice per week to determine the tumor onset and tumor size was determined using a caliper. When tumors reached 4 cm^3^ animals were sacrificed.

### Flow cytometry analysis

Lungs and livers from VE-Cadh^Snail1CT^ and VE-Cadh^Snail1KO^ mice or PyMT-VE-Cadh^Snail1CT^ and PyMT-VE-Cadh^Snail1KO^ tumors were collected in PBS and minced into 1 mm^3^ pieces. Afterwards, an enzymatic disaggregation was performed with the Tumor Dissociation Kit (MACS Miltenyi Biotec, 130-096-730) at 37 ºC for 30 min. A more exhaustive tissue disruption was made by gentleMACS Dissociator (MACS Miltenyi Biotec, 130-093-235). RBC Lysis Buffer (ThermoFisher, 00-4333) incubation was used to remove red blood cells. Cells were blocked with PBS supplemented with horse serum (20%) and FBS (10%) during 1 h at 4 ºC. Primary antibodies listed in Table S1 and specified for FACS were diluted in blocking solution and incubated during 40 min at 4 °C. DAPI staining was used as viability marker. Epithelial, endothelial and immune cells were separated from the rest of tumor stromal cells using the following gating: EpCAM+/CD45- were considered epithelial cells; EpCAM-/CD45+, immune cells; EpCAM-/CD45-/CD31+, endothelial cells and EpCAM-/CD45-/CD31-, other stromal cells. Cell staining was analyzed in a BD LSR II cytometer and data was processed using BD FACSDIVA software at the PRBB-FACS facility.

### *In vivo* Matrigel plug angiogenesis assay

VE-Cadh^Snail1CT^ and VE-Cadh^Snail1KO^ mice were subcutaneously injected in their flanks with 200 µL of Matrigel alone, or containing VEGFA (250 ng/mL) or FGF2 (1 µg/mL). One week later, mice were euthanized and Matrigel plugs were recovered and embedded in paraffin for analysis. Two medial sections of each plug were stained with CD31 and Snail1 antibodies by immunohistochemistry, slides were scanned and CD31-positive area was measured using ImageJ software. Three independent experiments were performed and the mean of each group was represented as the percentage of CD31-positive area per total Matrigel plug area.

### Transmission electron microscopy

Tumors of different sizes from PyMT-VE-Cadh^Snail1CT^ and PyMT-VE-Cadh^Snail1KO^ mice were dissected and fixed with p-formaldehyde (2%) plus glutaraldehyde (2%) in 0.2 M cacodylate buffer. Samples were stored in cacodylate buffer until processing. After post-fixation with 2% osmium tetroxide, samples were dehydrated and embedded in Epon LX112 (Ladd Research Industries). Semi-thin sections (approximately 1 μm-thick) were cut and stained with toluidine blue. Ultrathin sections (60-80 nm) were obtained in an ultramicrotome, placed on parlodion/carbon-coated nickel grids and stained with lead citrate and uranyl acetate. The grids were examined using a Phillips CM100 electron microscope.

### Epithelial tumor cell isolation from MMTV-PyMT tumors and orthotopic transplantation

Tumors from MMTV-PyMT female mice were sliced into 1 mm^3^ pieces and enzymatically disaggregated with the Tumor Dissociation Kit during 30 min at 37 ºC. Afterwards, RBC Lysis Buffer (ThermoFisher, 00-4333) was used to remove red blood cells. Tumor cells were filtered through a 70 µm-pore filter and individualized cells were plated in EpiCult-B culture media (Stemcell Technology, 05610) supplemented with murine EGF (10 ng/mL), FGF2 (10 ng/mL) and FBS (2%). One day later the culture medium was replaced by EpiCult-B culture media with EGF and FGF2. Tumoral cells (designed as ePyMT) were maintained without passing them and were inoculated during the first week after isolation.

Six weeks-old C57-Bl6 VE-Cadh^Snail1CT^ and VE-Cadh^Snail1KO^ female mice were inoculated intraperitoneally with Tamoxifen to delete Snai1 gene. Three weeks later, mice were anesthetized with isofluorane and both inguinal mammary fat pads were injected with 0.5 x 10^6^ ePyMT cells embedded in Matrigel. When tumors arrived to 4 cm^3^ (approximately 15 weeks), they were resected and processed for histological analysis.

## Results

### Snail1 is expressed in endothelial tumor cells

**Figure 1:**
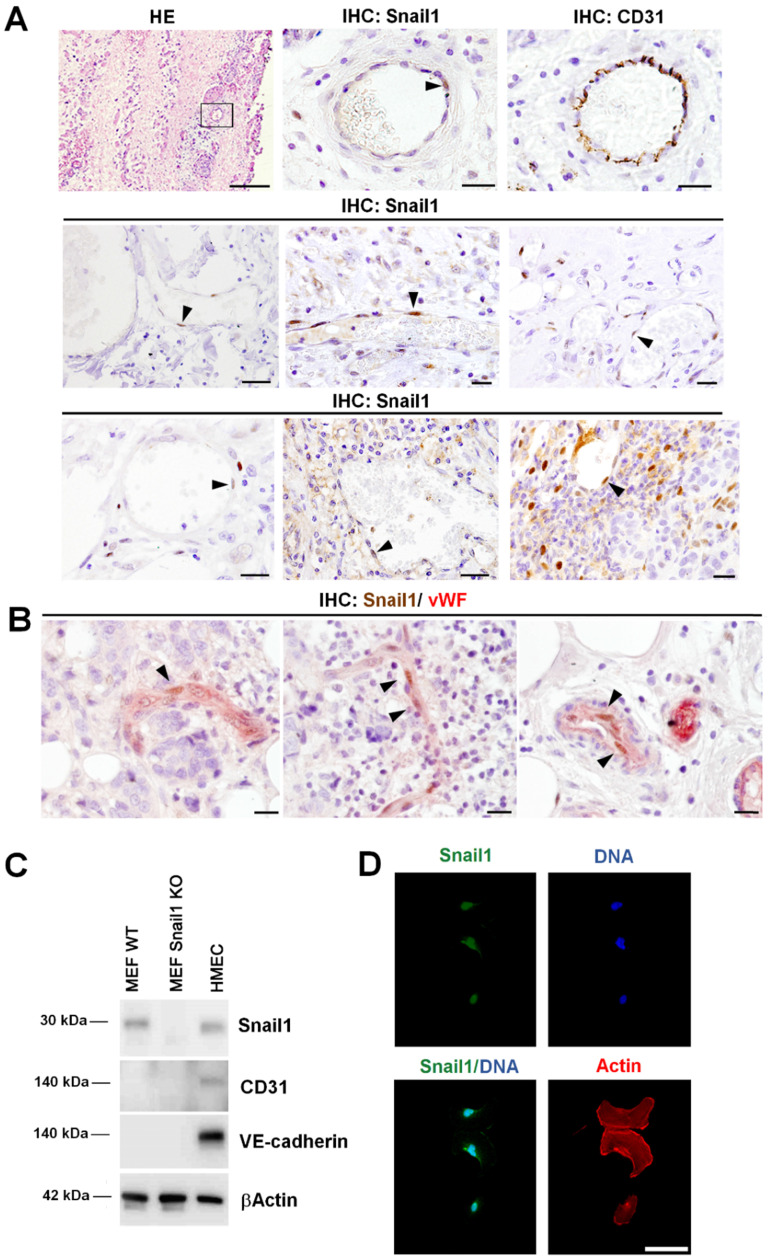
**Snail1 is expressed in human tumor endothelial cells**. **A,** Representative images of hematoxylin and eosin staining (HE) and Snail1 and CD31 immunohistochemistry in blood vessels of human neoplasms: colorectal carcinomas (first row), pancreatic adenocarcinomas (second row) and human fibromatosis (third row). Black arrowheads point Snail1+ cells. Scale bars: 100 µm (HE) and 20 µm (others). **B**, Double immunohistochemical staining of human breast tumors with Snail1 (brown) and the endothelium-specific protein von Willebrand factor (vWF) (red). Black arrowheads indicate examples of Snail1+ cells. Scale bar: 25 µm. **C**, Western blot analysis of Snail1 in HMEC-1 cells. MEF Wild-Type and KO for Snail1 were used as control. **D**, Snail1 (green) immunofluorescence analysis in cultured HMEC-1 cells. Actin cytoskeleton was stained with Alexa 555-conjugated Phalloidin (red) and nuclei were counterstained with DAPI (blue). Scale bar: 25 µm.

We have previously analyzed Snail1 expression in several human neoplasms, among them, pancreas and breast adenocarcinomas, colorectal carcinomas and fibromatosis 13, 23-25. In these tumors, Snail1 expression was observed mainly in CAF and in tumor cells placed in the invasive front. However, we also detected Snail1 expression in cells surrounding blood vessels 13, see also Figure 1A. These cells showed co-staining with a classical endothelial marker, CD31 (Figure 1A), suggesting that they are indeed endothelial cells. However, due to the close spatial relation between endothelial cells and pericytes, we wanted to assure this finding. We purified pericytes and endothelial cells from murine tumors by FACS (Figure S1A). Indeed, both types of cells exhibited similar levels of Snail1 mRNA as determined by qRT-PCR (Figure S1B). To further demonstrate Snail1 expression in endothelial cells we performed a double immunohistochemical analysis of Snail1 and von Willebrand factor (vWF), an endothelial marker. Presence of Snail1 in vWF-positive endothelial cells was detected in human breast tumors (Figures 1B and S1C). Interestingly, we never detected Snail1 in mature blood vessels from non-tumorigenic tissues (Figure S1D and see below). These results suggest that Snail1 might be required for angiogenesis, a process that does not take place in healthy adult animals.

Snail1 was detected in human microvasculature endothelial cells (HMEC-1) in culture at similar levels than in MEF (Figure 1C). Snail1 was mainly localized in the nucleus (Figure 1D) and was stimulated by VEGFA, a classical angiogenic factor (Figure S2A).

### Snail1 controls endothelial cell invasion and tubulogenesis

When plated on Matrigel, HMEC-1 cells align, branch and form a tubular polygonal network resembling a honeycomb, pattern characteristic of blood vessels. Snail1 protein was up-regulated during this process (Figure S2B). In order to investigate the role of Snail1 in these cells, we ectopically transfected a specific Snail1 siRNA (Figure S2C). Depletion of Snail1 caused a defective tubulogenesis, with a lower number of master junctions and master segments (Figures S2D and E). We also determined the relevance of Snail1 for invasion of HMEC-1 spheroids embedded in Collagen gels. HMEC-1 with down-regulated Snail1 displayed fewer invasive tips either in basal conditions and particularly, when stimulated with VEGFA or FGF2 (Figure S2F). These results suggest that Snail1 is essential for endothelial cell activation.

### *In vivo* endothelial cell invasion is dependent on Snail1

**Figure 2:**
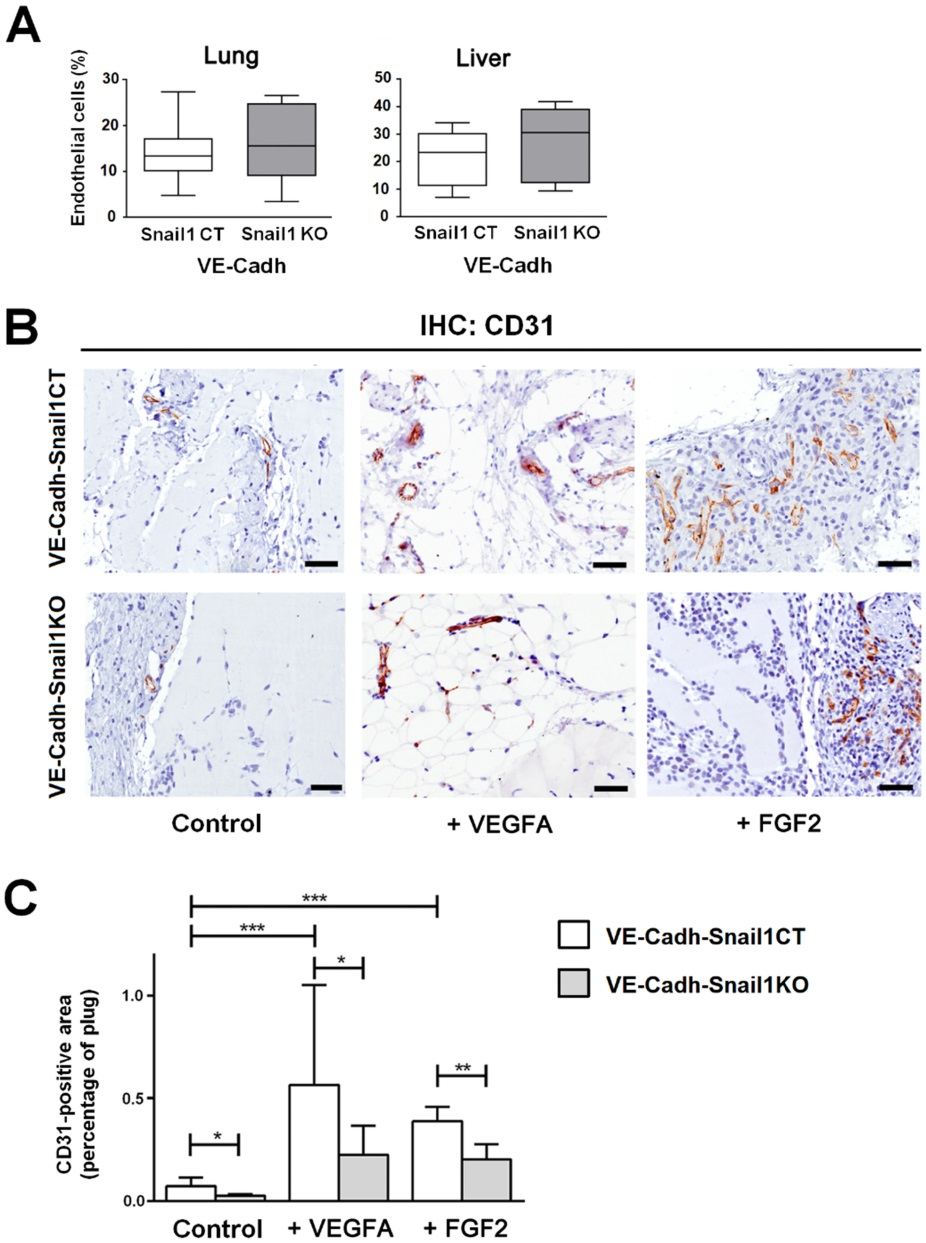
**Snail1 controls angiogenesis *in vivo***.** A**, Number of endothelial cells in lungs or livers of VE-Cadh^Snail1KO^ or VE-Cadh^Snail1CT^ mice. **B-C**, Matrigel plugs containing vehicle, VEGFA or FGF2 were xenografted in the flanks of VE-Cadh^Snail1CT^ or VE-Cadh^Snail1KO^ mice and analyzed one week later. B, Representative results of CD31 immunohistochemical analysis. Scale bar: 100 µm. C, Quantification of CD31 staining in Matrigel plugs. Data represent the mean ± SEM of three independent experiments. * P < 0.05; ** P < 0.01; *** P < 0.001.

Once we checked the relevance of endothelial Snail1 *in vitro*, we moved to an *in vivo* model. We generated a murine line with a specific depletion of this gene in the endothelium. To carried out the Snail1 deletion in this tissue, we used a Cre-ERT2 fusion protein under the control of the endothelium-specific Cdh5 (VE-Cadh) promoter 22. We generated mice carrying VE-Cadh-CreERT2, Snail1Flox and Snail1del (hereafter VE-Cadh^Snail1KO^) or Snail1 WT (VE-Cadh^Snail1CT^) and treated them with Tamoxifen when they were adult (six-weeks old) to promote an endothelium-specific depletion of Snail1 (Figure S3A). Isolated endothelial cells from VE-Cadh^Snail1KO^ mice showed a complete elimination of the floxed allele (Figures S3B and C). An aspect to remark is that Snail1 protein was not detected by immunohistochemistry in endothelial cells neither in VE-Cadh^Snail1CT^ nor in the VE-Cadh^Snail1KO^ animals (Figure S3D). In fact, VE-Cadh^Snail1KO^ mice do not show any overt phenotype and the size and morphology of their organs were not altered (Figures S3E and F). Moreover, the number of endothelial cells obtained from livers or lungs from VE-Cadh^Snail1KO^ animals was not different from controls (Figure 2A).

To test *de novo* angiogenic ability of those murine lines, we carried out an *in vivo* angiogenesis assay subcutaneously injecting Matrigel plugs supplemented or not with VEGFA or FGF2. Angiogenesis was determined analyzing by immunohistochemistry the number of endothelial cells inside the plug a week later. The number of CD31-positive endothelial cells in the plugs was stimulated by both FGF2 and VEGFA and was higher in VE-Cadh^Snail1CT^ mice than in VE-Cadh^Snail1KO^ mice (Figures 2B and C), demonstrating that angiogenesis was compromised by Snail1 depletion *in vivo*.

### Snail1 depletion in endothelial cells affects the onset of MMTV-PyMT breast tumors

**Figure 3:**
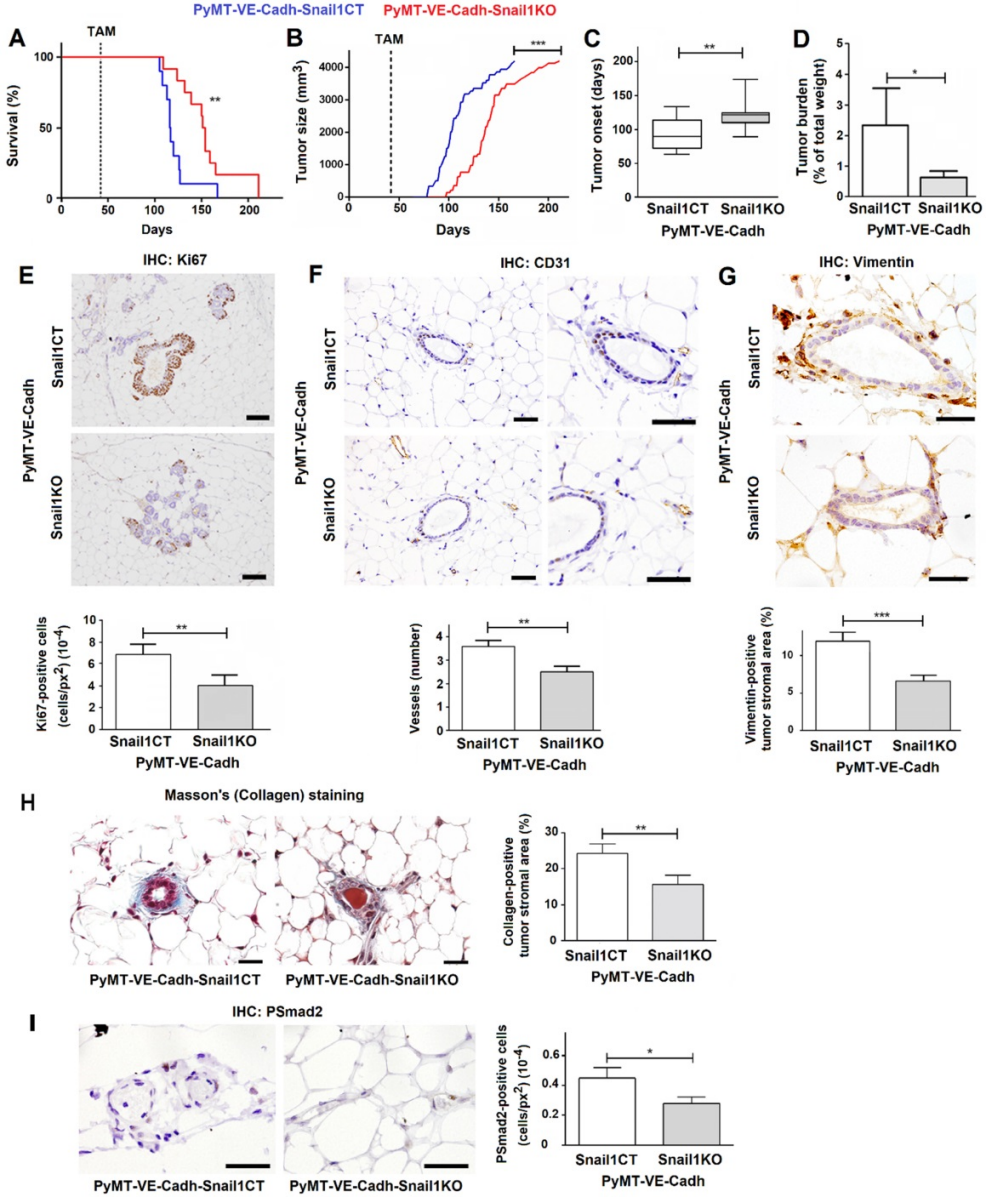
**Snail1 depletion in endothelial cells retards tumor onset and stromal activation in the MMTV-PyMT breast cancer model**. Comparison of survival (**A**), tumor size (**B**), tumor onset (**C**) and tumor burden at 18 weeks (**D**) between PyMT-VE-Cadh^Snail1CT^ and PyMT-VE-Cadh^Snail1KO^ mice. **E-I**, Representative images of Ki67 (**E**), CD31 (**F**), Vimentin (**G**), Masson's trichromic (**H**) or phosphoSmad2 (**I**) stainings of PyMT-VE-Cadh^Snail1CT^ and PyMT-VE-Cadh^Snail1KO^ at hyperplasic (E) or premalignant ducts (F-I). The quantification of the stainings was carried out as indicated in Suppl. Methods and is presented below or at the right. Bars correspond to 20 µm (CD31), 40 µm (Ki67, Vimentin and PSmad2) or 25 µm (Masson's). Data represent mean ± SEM of at least three mice per group. * P < 0.05; ** P < 0.01; *** P < 0.001.

Afterwards, we proceeded to analyze the effect of Snail1 endothelial depletion in breast tumor development mating our VE-Cadh-CreERT2, Snail1Flox/Snail1del (or Snail1Flox/Snail1WT) animals with mice expressing PyMT under the control of MMTV promoter (MMTV-PyMT mice). By eight to ten weeks these murine females spontaneously develop mammary gland tumors that progress to poorly differentiated invasive ductal carcinomas 19, 26. PyMT-VE-Cadh^Snail1CT/KO^ mice were treated with tamoxifen at six weeks of age, to eliminate Snail1 in the endothelium, and tumor development was monitored. Mice depleted of Snail1 in the endothelium presented a greater survival with respect to controls (Figure 3A). They also showed a delayed tumor growth and a later tumor onset (Figures 3B and C); at 18 weeks, the time that PyMT-VE-Cadh^Snail1CT^ mice had to be euthanized, the tumor burden was higher in these animals than in PyMT-VE-Cadh^Snail1KO^ mice (Figure 3D). Snail1 was detected mainly in CAF (Figure S4A), although some endothelial cells also presented Snail1 expression in PyMT-VE-Cadh^Snail1CT^ animals (Figure S4B). Snail1 RNA expression was remarkably inhibited in endothelial cells from PyMT-VE-Cadh^Snail1KO^ tumors (Figure S4C).

We investigated the cause of the striking difference in the tumor onset. The initial preneoplasic lesions showed a lower proliferation in mice with endothelial-specific Snail1 depletion compared with control mice as they displayed less Ki67 expression (Figure 3E). These ducts showed a reduced angiogenesis in PyMT-VE-Cadh^Snail1KO^ animals and fewer vessels, reinforcing the idea of a defective invasion of endothelial cells (Figure 3F). Moreover, premalignant ducts presented a lower activation of fibroblasts in PyMT-VE-Cadh^Snail1KO^ mice, as assessed analyzing the expression of the fibroblast activation marker Vimentin (Figure 3G) or determining Collagen stromal deposition by Masson's trichromic staining (Figure 3H). This result suggests that Snail1 expression in endothelial cells, besides participating in angiogenesis, also facilitates CAF activation.

We validated these results *in vitro*, determining the capability of conditioned medium derived from HMEC-1 cells to activate fibroblasts. Conditioned medium from control HMEC-1 cells promoted a higher increase in the expression of fibroblast activation markers such as Snail1, Vimentin and Fibronectin in MEF than when these cells were cultured with conditioned medium from Snail1-depleted HMEC-1 cells (Figure S5A). Conditioned medium from Snail1-depleted cells was also less capable to stimulate the expression of *Vim*, *Col1A1, Col1A2, Tgfb1, Tgfb2* and *Fgf2* in MEFs (Figure S5B), all genes upregulated upon fibroblast activation.

In order to find the secreted factor responsible for this activation, we analyzed the conditioned medium from HMEC-1 siControl and siSnail1 with a cytokine and growth factor antibody array. Snail1 down-regulation affected the HMEC-1 secretion of several factors such as TGFβ2 and others (Figure S5C). We validated this *TGFB* decreased expression by qRT-PCR and extended it to other fibroblast activating factors not analyzed in the array (Figure S5D). In accordance with their well-known effect on fibroblast activation, inhibition of TGFβ with SB505125 (SB) 27 decreased the stimulation of different markers of active fibroblast by the conditioned medium of HMEC-1 cells (Figure S5E); the tyrosine kinase inhibitor Crenolanib (CRN) 28 also caused a similar effect. Therefore, these results indicate that endothelial cells modulate fibroblast activation through the Snail1-dependent production of TGFβ and probably other factors. Accordingly, neovessels close to premalignant ducts from PyMT-VE-Cadh^Snail1CT^ showed a higher number of phosphoSmad2-positive cells than those from PyMT-VE-Cadh^Snail1KO^, a parameter that was used as a read-out for *in vivo* TGFβ secretion (Figure 3I).

### Snail1 depletion in endothelial cells modifies MMTV-PyMT tumor phenotype

**Figure 4:**
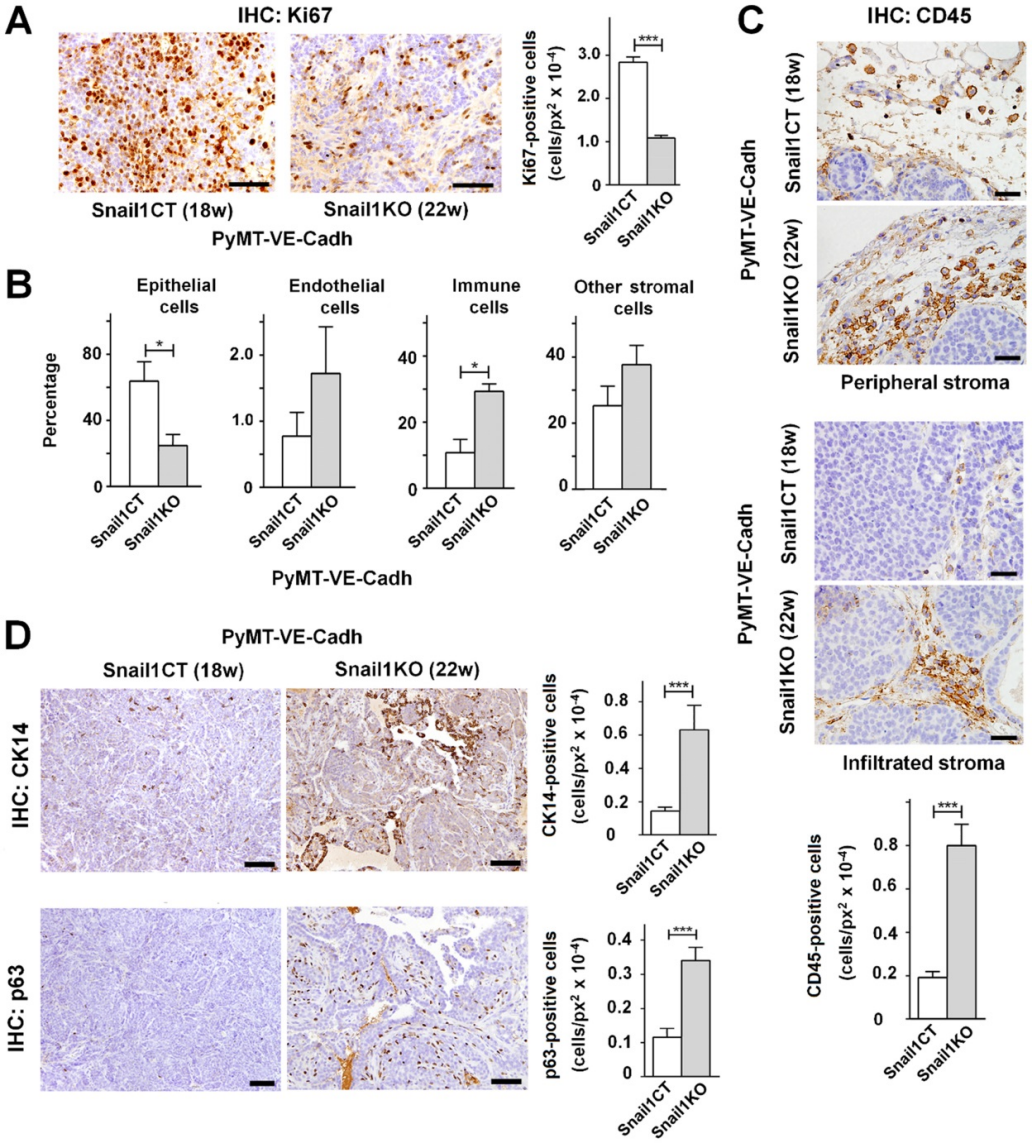
**Endothelium-specific Snail1 depletion alters tumor cell content of MMTV-PyMT tumors**. **A**, Immunohistochemical Ki67 analysis (images, left panel; and quantification, right panel) of PyMT-VE-Cadh^Snail1CT^ and PyMT-VE-Cadh^Snail1KO^ carcinomas at 18 and 22 weeks, respectively. Scale bar: 40 µm. **B**, Percentage of epithelial, endothelial, immune and other stromal cells in PyMT-VE-Cadh^Snail1CT^ and PyMT-VE-Cadh^Snail1KO^ carcinomas determined by FACS. **C-D**, CD45 (C), CK14 and p63 (D) analysis (of the indicated carcinomas. Scale bar: 40 µm (C); 200 µm (D). In the quantifications, data represent mean ± SEM of at least three mice per group. * P < 0.05; *** P < 0.001.

Apart from tumor onset, differences were also found in tumor cellular composition. At the time of sacrifice, 18 weeks for PyMT-VE-Cadh^Snail1CT^ mice and 22 weeks for PyMT-VE-Cadh^Snail1KO^ mice, tumors with endothelial-specific Snail1 depletion showed a lower epithelial proliferation as assessed by Ki67 staining (Figure 4A). When analyzed by FACS at the end-point, the cellular composition of the tumors was different as PyMT-VE-Cadh^Snail1KO^ mice presented a lower presence of epithelial cells whereas the percentage of immune cells was higher compared to PyMT-VE-Cadh^Snail1CT^ mice (Figure 4B). The higher infiltration of immune cells in PyMT-VE-Cadh^Snail1KO^ mice was also verified by immunohistochemistry not only in the peripheral stroma, but also intratumorally (Figure 4C). Moreover, PyMT-VE-Cadh^Snail1KO^ mice included a higher number of myoepithelial cells characterized by the expression of p63 and K14 (Figure 4D).

**Figure 5:**
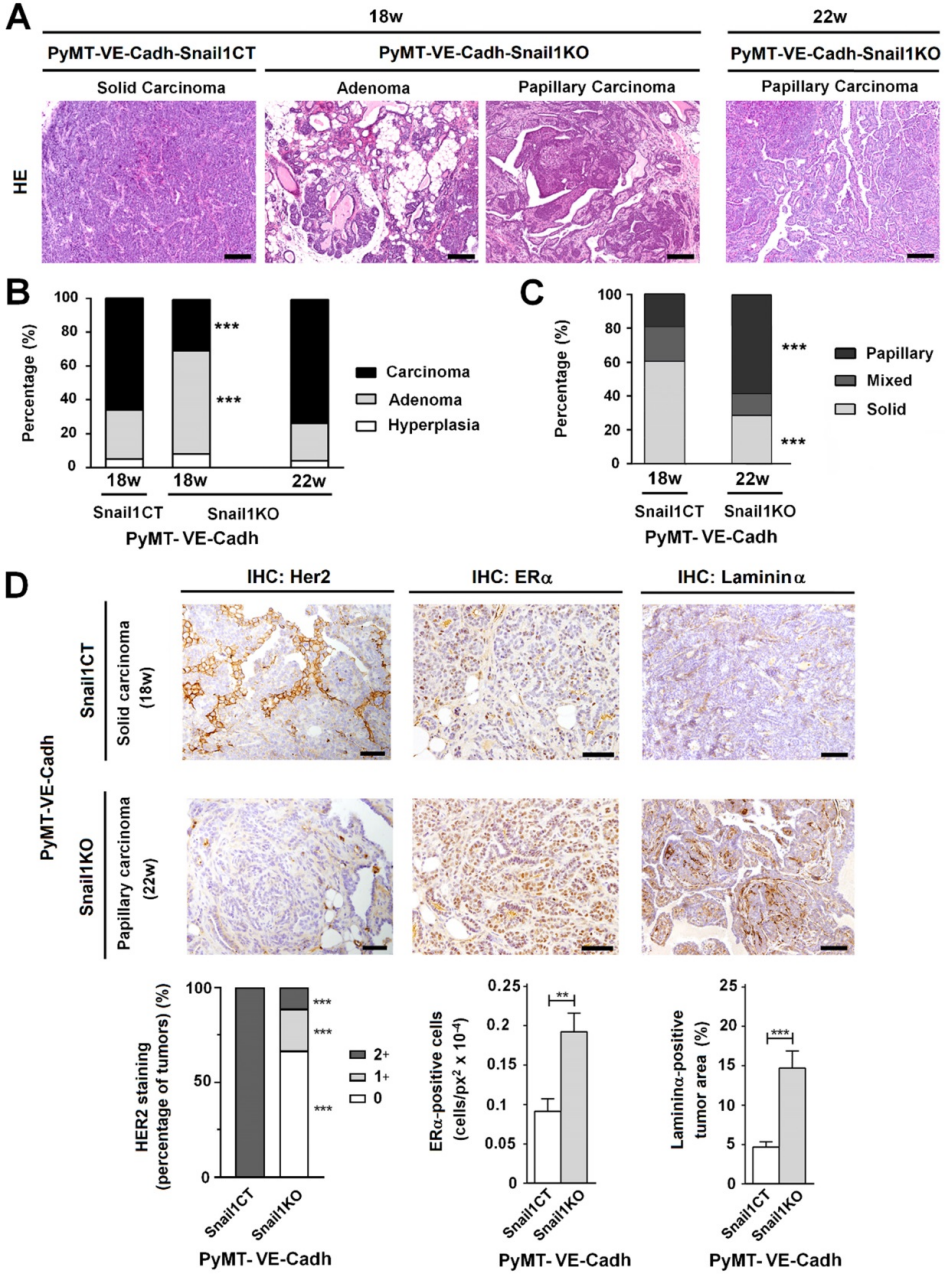
**Tumors with an endothelial-specific Snail1-depletion show an alteration in tumor differentiation. A**, Hematoxylin and eosin staining of tumors obtained from PyMT-VE-Cadh^Snail1CT^ and PyMT-VE-Cadh^Snail1KO^ mice at the indicated time points. Scale bars: 200 µm. **B-C**, Quantification of tumor stage (B) and carcinoma morphology (C) in PyMT-VE-Cadh^Snail1CT^ and PyMT-VE-Cadh^Snail1KO^ tumors obtained at the indicated time points. **D**, Her2, ERα and Laminin α analysis of carcinomas generated in PyMT-VE-Cadh^Snail1CT^ and PyMT-VE-Cadh^Snail1KO^ mice. Scale bar: 50 µm. Staining quantification was performed as indicated in the Supplemental Information and is presented below. ** P < 0.01; *** P < 0.001.

The morphology of the tumors was also strikingly different. At 18 weeks, when most of PyMT-VE-Cadh^Snail1CT^ mice had to be euthanized, tumors displayed features of a solid invasive carcinoma (Figure 5A-C). In contrast, in PyMT-VE-Cadh^Snail1KO^ mice, most of the tumors showed characteristics of adenomas at this time. Four weeks later, when the vast majority of PyMT-VE-Cadh^Snail1KO^ mice were sacrificed, these mammary gland tumors had also progressed to carcinomas (Figures 5A and B). However, compared to tumors in PyMT-VE-Cadh^Snail1CT^, mostly solid carcinomas, PyMT-VE-Cadh^Snail1KO^ carcinomas were mainly papillary (Figure 5C). An analysis of these carcinomas showed that PyMT-VE-Cadh^Snail1CT^ tumors exhibited a higher HER2 expression with respect to PyMT-VE-Cadh^Snail1KO^ tumors, whereas estrogen receptor expression was retained at a greater level in PyMT-VE-Cadh^Snail1KO^ tumors (Figure 5D). Neoplasms generated in PyMT-VE-Cadh^Snail1KO^ mice presented lower tumoral invasion as determined by the staining of the basement membrane component Laminin α (Figure 5D) 29. Tumor necrosis was higher in PyMT-VE-Cadh^Snail1KO^ carcinomas (Figures S6A and B). This was not associated to a different necrosis status depending on the morphology of the tumors, since solid versus papillary carcinomas obtained from PyMT-VE-Cadh^Snail1CT^ animals did not show differences in this parameter (Figure S6B). In summary, these results show that endothelial-depletion of Snail1 derives in more differentiated tumors.

### Snail1 depletion in endothelial cells alters MMTV-PyMT tumor angiogenesis

**Figure 6:**
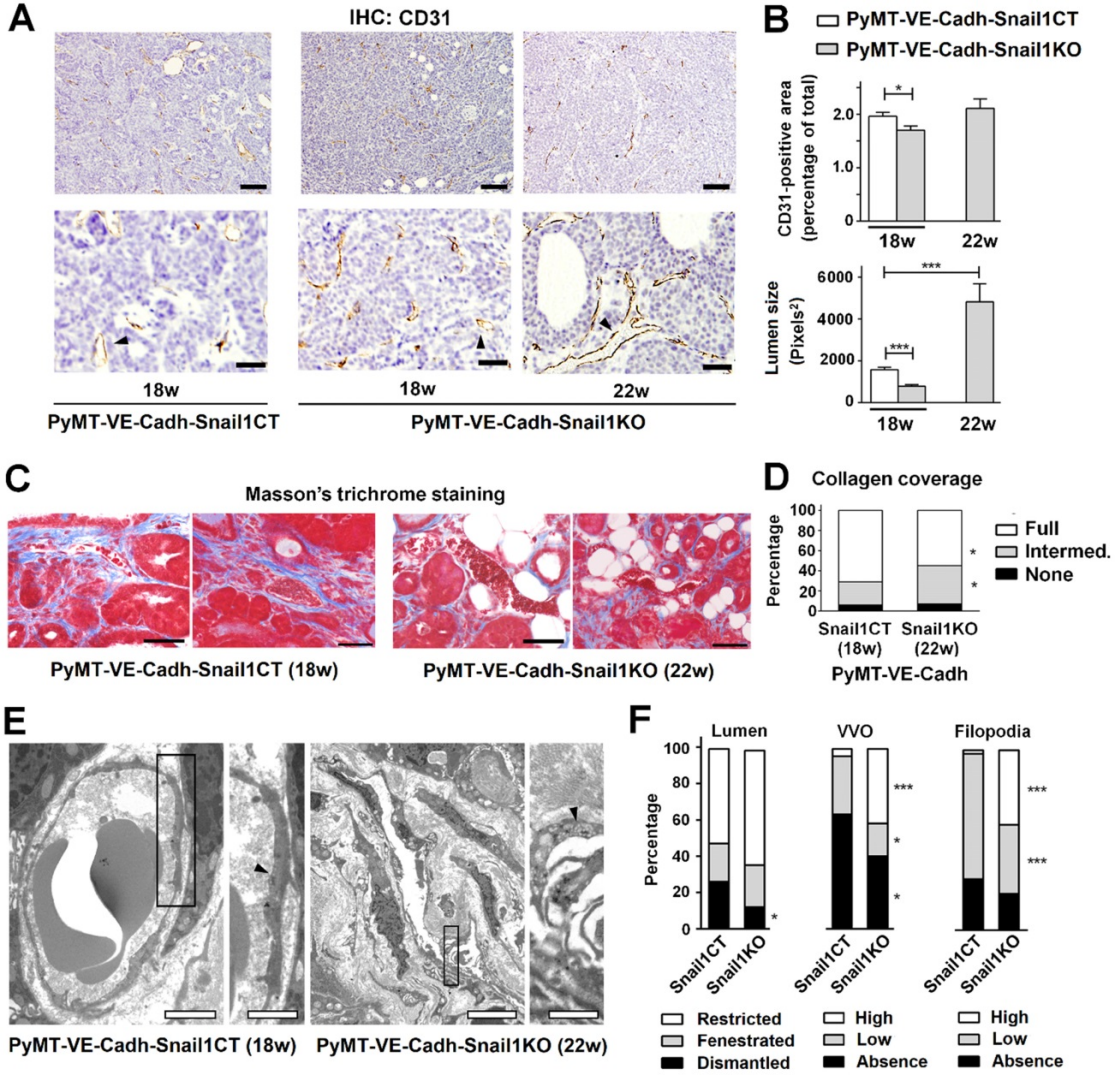
**Alterations in tumor vasculature associated to Snail1 depletion**. **A**, CD31 analysis of PyMT-VE-Cadh^Snail1CT^ and PyMT-VE-Cadh^Snail1KO^ tumors collected at the indicated times. Scale bars: 200 µm (upper row); 40 μm (bottom row). **B**, Quantification of CD31+ area (upper graph) and vessel lumen size (bottom graph). **C**, Masson's trichrome staining of MMTV-PyMT tumors. Scale bar: 50 μm. Data in B and C represent mean ± SEM of at least three independent experiments. **D**, Quantification of vessel Collagen coverage as the percentage of vessels displaying full, intermediate or absent coating by Collagen fibers. **E**, Transmission electron microscopy images of PyMT-VE-Cadh^Snail1CT^ and PyMT-VE-Cadh^Snail1KO^ tumor vessels. Arrowheads label vesiculo-vacuolar organelles (VVO) in the endothelial cells of the capillaries. Scale bars: 2 μm (PyMT-VE-Cadh^Snail1CT^ mice); 5 μm (PyMT-VE-Cadh^Snail1KO^ mice); 1 μm (magnifications of both). **F**, Quantification of vessel lumen integrity, number of VVO and presence of filopodia in MMTV-PyMT carcinoma vessels. More than fifty vessels of different tumors per group were analyzed. Data represents the percentage of the vessels at the defined categories. * P < 0.05; *** P < 0.001.

Tumor angiogenesis was also examined. PyMT-VE-Cadh^Snail1CT^ tumors showed a significant greater percentage of endothelial cells than PyMT-VE-Cadh^Snail1KO^ tumors at 18 weeks, when control mice had to be euthanized (Figures 6A and B). However, when PyMT-VE-Cadh^Snail1CT^ and PyMT-VE-Cadh^Snail1KO^ tumors were compared at the time of sacrifice (18 and 22 weeks, respectively), the percentage of endothelial CD31+ cells in the tumors was similar (Figure 6B), in accordance with the data previously presented in Figure 4B. However, vessels from these tumors exhibited differences in their morphology since those from endothelial Snail1-depleted mice (collected at 22 weeks) showed a wider lumen (Figures 6A and B). These vessels also presented less complete Collagen coverage compared to controls (Figures 6C and D), suggesting that are less capable to invade Collagen matrices, in accordance with our results obtained *in vitro* with HMEC-1 spheroids (see Figure S2F).

Blood vessels from tumors collected at 18 weeks in PyMT-VE-Cadh^Snail1CT^ animals or 22 weeks in PyMT-VE-Cadh^Snail1KO^ mice were studied by transmission electronic microscopy. Capillaries from both groups presented a high degree of morphological anomalies likely due to the pro-angiogenic environment sustained by tumoral cells. However, when both groups were compared, PyMT-VE-Cadh^Snail1KO^ tumors exhibited a better maintenance of the lumen integrity without fenestrae or ruptures, a higher presence of vesiculo-vacuolar organelles and a greater abundance of intraluminal protrusions (Figures 6E and F), features that have been related to wider vessels (see Discussion).

### Alterations in tumor phenotype are also consequence of the interference in tumor angiogenesis

In order to reproduce these results, we orthotopically grafted epithelial tumor cells obtained from PyMT carcinomas (ePyMT cells) in syngeneic VE-Cadh^Snail1CT^ or VE-Cadh^Snail1KO^ mice mammary glands. Injection of a high number of cells originated tumors in both animals with the same latency (Figure S7A). The morphology of the tumors was different, reproducing the results obtained with spontaneous tumors. Although in both backgrounds ePyMT created carcinomas, at the time of sacrifice these were mostly solid in VE-Cadh^Snail1CT^ mice whereas in VE-Cadh^Snail1KO^ they were papillary or mixed carcinomas, with both papillary and solid areas (Figures S7B-D). Tumors generated in VE-Cadh^Snail1KO^ mice also showed less angiogenesis and vessels with a wider lumen (Figures S7E and F), as the spontaneous MMTV-PyMT model (see above).

**Figure 7:**
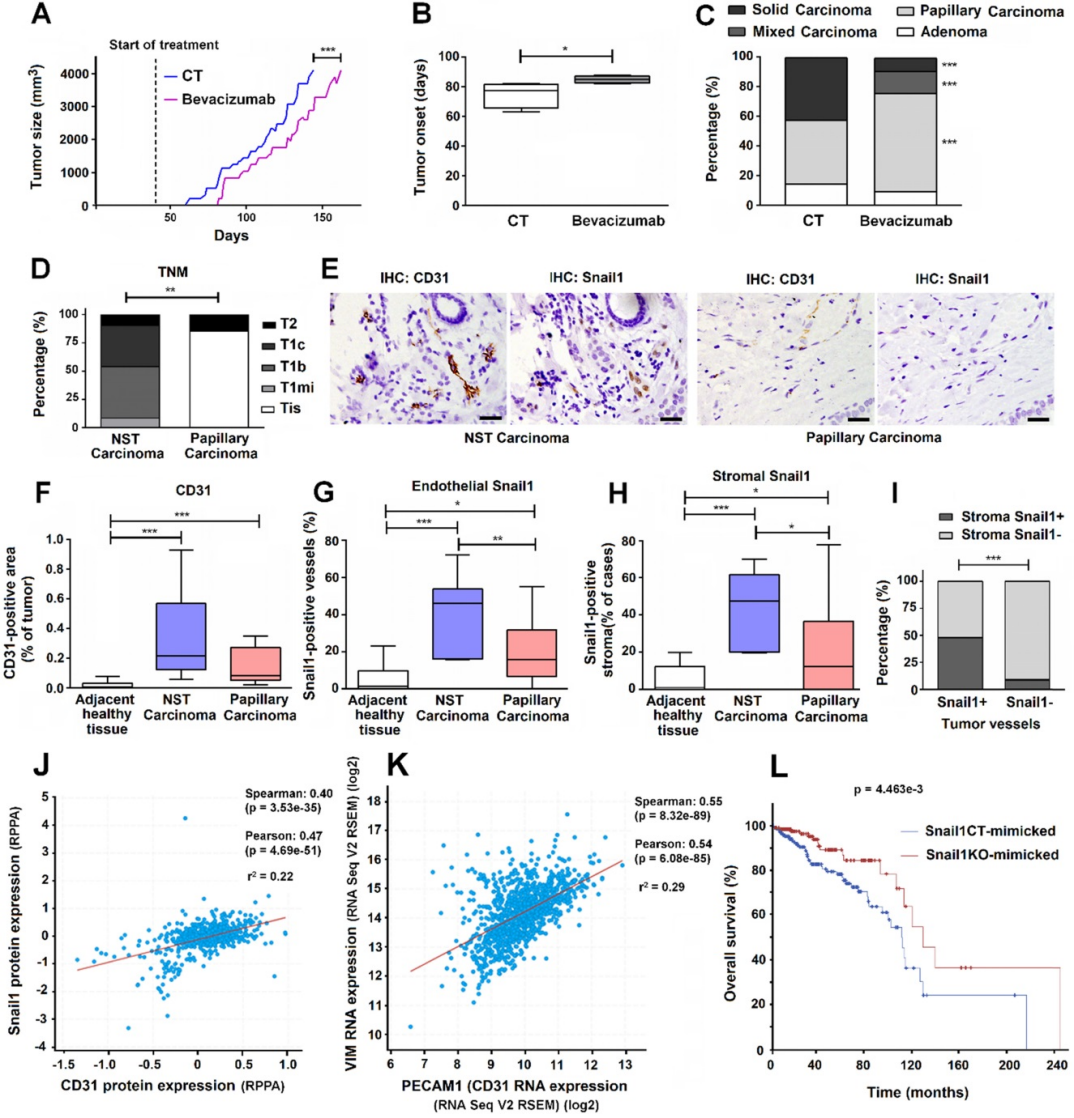
**Reduced angiogenesis is associated to papillary carcinoma morphology in a pre-clinical murine model and human breast tumor samples**. **A-C**, Tumor size (A), tumor onset (B) and carcinoma morphology (C) of MMTV-PyMT mice treated with Bevacizumab. **D**, Tumor stage in a cohort of patients with non-specific type (NST) or papillary breast carcinomas. Data are represented as percentage of cases according to breast cancer TNM classification. **E-I**, Analysis of CD31 and Snail1 expression in breast tumors; representative images of the immunochemical staining of an NST and a papillary carcinoma are presented in E. Bars in E corresponds to 100 μm. The quantification of CD31+ areas per tumor case (F), the percentage of tumor vessels expressing Snail1 per tumor case (G) and the percentage of Snail1+ stromal areas in the vicinity of vessels per tumor cases (H) was represented in normal breast tissue adjacent to tumors, NST carcinomas and papillary carcinomas. I, Percentage of human tumor blood vessels expressing or not Snail1 with Snail1+ stromal cells in the vicinity. **J-K**, Regression analysis of CD31 protein versus Snail1 protein expression (J) and PECAM1 (CD31) mRNA versus VIM mRNA expression (K) in human breast cancer tumor samples from TCGA consortium data. Regression lines between the indicated parameters and corresponding r2 goodness-of-fit values are shown for each graph, as well the Pearson and Spearman correlation coefficients with their corresponding P value. **L**, Kaplan-Meier survival curves of patients with breast tumors mimicking those obtained in PyMT-VE-Cadh^Snail1CT^ and PyMT-VE-Cadh^Snail1KO^. Log rank test P value is shown in the graph. * P < 0.05; ** P < 0.01; *** P < 0.001.

Since Bevacizumab has been successfully used for inhibiting VEGF signaling in immunocompetent mice 30-33, we used this tool to mimic our results of Snail1 depletion in the MMTV-PyMT breast tumor model. We started the treatment with this drug at the same day that Snail1 was depleted by Tamoxifen injection in PyMT-VE-Cadh^Snail1CT^ or PyMT-VE-Cadh^Snail1KO^ mice, much earlier than tumors were detectable. Bevacizumab significantly retarded tumor onset (Figures 7A and B). Moreover, Bevacizumab treated-mice also presented a much higher proportion of papillary carcinomas than control mice (Figure 7C). At the day of sacrifice, the number of endothelial cells was not significantly different but vessels in Bevacizumab-treated tumors exhibited a higher lumen (Figure S8), alike we have shown for tumors developed in endothelial Snail1-depleted mice.

### Human papillary breast carcinomas show lower expression of endothelial and stromal Snail1 than other types of breast tumors

Finally, in order to reinforce our results, we also analyzed a small cohort of human breast papillary carcinomas that we compared with other breast neoplasms, as a representation of PyMT-VE-Cadh^Snail1CT^ and PyMT-VE-Cadh^Snail1KO^ carcinomas, respectively. As shown in Figure 7D, at the time of diagnosis, papillary carcinomas presented a lower grade than the rest of tumors (not-specified type, NST carcinomas). They also showed lower angiogenesis (Figures 7E and F) and lower staining of Snail1 both in endothelial cells (Figures 7E and G) and other stromal cells, mostly fibroblast-alike cells (Figure 7E and H). Expression of Snail1 in endothelial cells and in fibroblast cells was strongly associated since tumors that showed Snail1 expression in tumor-associated vessels also exhibited a higher presence of this protein in fibroblasts (Figure 7I).

Using a large human breast tumor database, we observed that CD31 protein levels correlated with those of Snail1 in breast tumor datasets (Figure 7J). The TCGA database used also showed a significant positive correlation between endothelial cells (as identified by PECAM1 (CD31) RNA expression) and CAF activation (assessed by VIM (Vimentin) RNA) (Figure 7K). Finally, we created two humanized signatures from PyMT-VE-Cadh^Snail1CT^ and PyMT-VE-Cadh^Snail1KO^ tumors, based on our findings in mice, to check their prognosis in humans. Compared to controls, tumor displaying lower SNAI1, PECAM1 and VIM and higher TP63, KRT14, KRT5 and PTPRC (CD45) RNAs (PyMT-VE-Cadh^Snail1KO^ tumor signature) presented a longer overall survival (Figure 7L), strengthen our mice results.

## Discussion

Snail1 expression has been observed in different types of epithelial tumors mainly in CAF 23, 34. Since it reflects the activation of these cells, Snail1 presence in CAF associates to a worse prognosis in breast tumors 25 among other neoplasms. Moreover, besides being present in invasive epithelial cells and CAF, we show here that Snail1 is also detected in endothelial tumor cells. Specific depletion of Snail1 in these cells exerts remarkable consequences in tumor development and progression.

First, it delays tumor onset. This effect was associated to a lower proliferation of the initial lesions and to a lower activation of the associated stroma. Regarding the crosstalk between endothelial and mesenchymal cells, we have determined that expression in HMEC-1 of several factors capable to activate CAF, such as TGFβ2 and PDGF-C, is sensitive to Snail1 depletion. Therefore, we suggest that at the initial phases of tumorigenesis the activated endothelium is crucial for the stimulation of mesenchymal cells from the stroma, likely cooperating with other signals derived from epithelial tumor cells. It is possible that this effect is more relevant at the initial phases of tumor growth when the number of transformed cells is still low, and not at later times when the cytokines derived from tumor cells are probably sufficient to activate the stroma by themselves.

We also observed a delayed progression of the tumors. When we matched tumors of similar size, those generated in mice with a Snail1 deficiency in endothelial cells presented a lower proliferation associated to a higher presence of myoepithelial cells. Myoepithelial cells encircle ducts and acini of glands, creating a natural border separating proliferating epithelial cells from basement membrane and underlying stroma; thus, physically preventing tumor cell invasion. Several lines of evidences 35 suggest that myoepithelial cells block proliferation of breast carcinoma cells by inducing growth arrest and apoptosis. Probably as a consequence of this higher content of myoepithelial cells, tumor progression is delayed and when PyMT tumors had advanced to carcinomas in Snail1 control mice, only adenomas were detected in endothelial Snail1-depleted animals.

Moreover, when we compared carcinomas from both mice groups, obtained at later times in PyMT-VE-Cadh^Snail1KO^ than in PyMT-VE-Cadh^Snail1CT^ mice, we observed that the carcinoma morphology was not the same in both models: whereas in control animals were mostly solid, invasive and poorly differentiated carcinomas, in endothelial Snail1-depleted mice, they were papillary, non-invasive and more differentiated carcinomas. In humans, these papillary tumors present a better prognosis than ductal carcinomas 36. Murine tumors generated under depletion of Snail1 in endothelial cells also presented more abundant areas of necrosis, suggesting that angiogenesis was affected. Blood vessels irrigating these tumors displayed morphological differences: compared with the vessels observed in tumors from control mice, thin and with reduced lumen, tumors from endothelial Snail1-depleted mice presented wider vessels. Ultrastructural studies also showed that their vascular cells displayed higher number of vesiculo-vacuolar organelles that have been associated to the formation of larger vessels 37. The number of filopodia, another structure also associated to greater vessels 38, was also increased in PyMT-VE-Cadh^Snail1KO^ tumors. Therefore, we suggest that Snail1-depleted vessels respond to pro-angiogenic cues, migrating in cohesive manner, with a lower capacity to supply nutrients to the total tumor volume; hence, originating more areas of necrosis.

Two other experimental models corroborated these conclusions. First, orthotopic xenografting of ePyMT tumor cells in a syngeneic murine model with a Snail1 genetic depletion in endothelial cells also showed carcinomas with a more papillary morphology than when the cells were injected in control mice. Moreover, the very early supplementation with Bevacizumab to MMTV-PyMT mice also reproduced the results obtained by Snail1 depletion. It is important to remark that these effects of Bevacizumab required its administration starting at the same moment we depleted Snail1 in our mice; thus, before tumors were initially detected. This is much earlier than the usual time of treatment with this drug in human patients, administrated to already established tumors, and it is probably related to a function for activated endothelial cells in tumoral onset. We also analyzed human breast tumors, confirming that, compared to papillary neoplasms, solid tumors displayed higher expression of Snail1 both in endothelial cells and in other stromal cells.

In summary, these results demonstrate that, in addition to its role in tumor cells and CAFs, during tumor progression, Snail1 also plays a very relevant action in endothelial cells, modulating tumor angiogenesis, tumor progression and prognosis. Moreover, they also uncover a new action of Snail1-dependent activated endothelial cells in tumor initiation through the paracrine stimulation of mesenchymal tumor stromal cells.

## Supplementary Material

Supplementary methods, figures and tables.

## Abbreviations

BSA: bovine seroalbumin
CAF: cancer associated fibroblast
CRN: crenolanib
DMEM: Dulbecco-modified Eagle's medium
EMT: epithelial-to-mesenchymal transition
ePyMT: epithelial tumor cells derived from MMTV-PyMT mice tumors
FBS: fetal bovine serum
HMEC-1: human microvascular endothelial cells 1
IHC: immunohistochemistry
MEF: mouse embryonic fibroblasts
NST carcinoma: not-specific type carcinoma
PyMT: Polyomavirus middle T antigen
SB: SB505125
TBS: tris buffered saline
WB: Western Blot
VVO: vesiculo-vacuolar organelles
vWF: von Willebrand factor
