# Supplementary material for: Snail1 expression in endothelial cells controls growth, angiogenesis and differentiation of breast tumors
Source: Theranostics. 2021 Jun 16;11(16):7671–84. doi: 10.7150/thno.61881 (PMC8315050; doi:10.7150/thno.61881)
Supplement: Supplementary file 1 — Supplementary methods, figures and tables. [file thnov11p7671s1.pdf]

Snail1-dependent activation of endothelial cells controls growth, angiogenesis and differentiation of breast tumors.

David Cabrerizo-Granados, Raúl Peña, Laura Palacios, Laura Carrillo-Bosch, Josep Lloreta-Trull, Laura Comerma, Mar Iglesias, and Antonio García de Herreros

SUPPLEMENTARY METHODS

Matrigel induced tubulogenesis assay. Fifty µl of Matrigel (Matrigel® Growth Factor Reduced, Corning, 354230) were polymerized at 37 ºC in 24-well plates. After 30 min, 1.1×10^5 HMEC-1 cells per well were plated in culture medium. Wells were imaged at indicated time points using a Leica DMIL Led microscope. Angiogenesis Analyzer plug-in for ImageJ was used for automatic quantification. Number of master segments (linear aggregates of endothelial cells between nodes) and master junctions (nodes connecting three segments) (see Figure S2D) were represented. Each experiment was repeated three independent times for statistical analysis.

Spheroid based sprouting assay. Spheroids were generated by the hanging drop technique. Briefly, 500 HMEC-1 were dispensed in 30 µL of carboxymethylcellulose (0.25%, w/v) in standard medium on the lid of non-adherent plates and incubated upside down overnight. Spheroids were embedded into Collagen type I (2 mg/mL) and cultured in standard medium. VEGFA (20 µg/mL) or FGF2 (100 ng/mL) were added to standard medium when indicated. Spheroids were fixed 24 h later with glutaraldehyde (3%) during 5 min at room temperature. Sprouting was quantified by measuring the number of sprouts per spheroid. A minimum of 10 spheroids per condition were analyzed in three independent experiments for statistical analysis.

Quantification of immunohistochemical analyses. For the quantification of Ki67-positive cells, ten premalignant lesions or carcinomas per mice (three animals per group) were micrographed and Ki67-positive epithelial nuclei were counted manually. The graph shows the mean of positive cells per breast epithelial area. To evaluate Vimentin staining, 10 premalignant lesions per mice (three per group) were imaged and stromal area was manually delimitated. Percentage of Vimentin-positive area within stroma was calculated with ImageJ [1] and represented. PhosphoSmad2 immunostaining was assessed in a similar way analyzing open vessels in the vicinity of the ducts. Cells expressing CD45 cells were manually counted in ten random images of the peripheral stroma and the infiltrated area of end-point tumors (three animals per group). Mean of the positive cells per area was represented. p63, CK14 and ERα-positive cells were manually counted in ten random images of carcinoma (three animals per group) and the mean of the positive cells per area was represented. For Lamininα, the percentage of Lamininα-positive area within the tumor was calculated with ImageJ and represented.

For Collagen quantification, Masson´s trichrome stains were scanned with Aperio ScanScope (Leica) at the Anatomy Department, Hospital del Mar, and a minimum of 5 premalignant lesions per group were acquired using QuPath software. Quantification was performed according to [2]. Briefly, blue coloration, indicative of collagen, was separated for all images by manual thresholding of hue (121-179), saturation (20-255), and brightness (10-255) values in ImageJ and the percentage of Collagen area in the stroma was represented.

Her2 protein expression was detected by IHC and quantified by expert pathologists by visual estimation of staining intensity comparing to appropriate controls. Each sample was scored and classified into four-tiered scale, depending its signal: 0 (no signal), 1+, 2+ and 3+ (maximum intensity).

Snail1 and CD31 were detected by IHC in consecutive slides from human breast tumors. Ten random areas were imaged at 10x magnification. CD31 stained area was measured using ImageJ software and microvessel density was quantified as the percentage of CD31-positive area per image area. Lumen size of the vessel was obtained by the software. Snail1 presence in the vessels or stroma was manually assessed in each area.

Reverse transcription and real-time quantitative PCR. Total RNA was extracted using GenElute TM Mammalian Total RNA Miniprep Kit (Sigma). Expression levels of transcripts were calculated by real-time quantitative PCR coupled to retrotranscription (RT-PCR), using the Transcriptor First Strand cDNA Synthesis kit (Roche) and the LightCycler 480 Real-Time PCR System (Roche). RNA levels were determined in triplicate. Reactions were performed using the primers listed in Table S2.

Cell lysis and protein analysis by western blot. Cell extracts were obtained in SDS lysis buffer (Tris-HCl pH 7.4, 50 mM; SDS 2% and glycerol 10%) and analyzed by western blot using the indicated primary antibodies (Table S1) and HRP-conjugated secondary antibodies.

Statistical analysis. Data were analyzed by GraphPad Prism (v6) software (GraphPad Software, San Diego, CA, USA). Survival graphs were represented by a Kaplan Meier curve and P value was obtained using log-rank test. Statistical comparisons of the quantifications of tumor histological subtypes, vessel collagen coverage, transmission electronic microscopy analyses, TNM and Snail1 presence in Snail1 vessels were performed by $\chi 2$ (and Fisher's exact) test. Graphs and statistical analyses from cBioportal datasets were obtained directly from their webpage. In all other experiments, statistical significance was obtained using Student's t-test; P value was represented in all the figures by * when P value < 0.05; **, when P value < 0.01; or ***, when P value < 0.001.

Supplementary References

1. Schindelin J, Arganda-Carreras I, Frise E, Kaynig V, Longair M, Pietzsch T, et al. Fiji: an open-source platform for biological-image analysis. Nat Meth. 2012; 9: 676-682.

2. Bauman TM, Nicholson TM, Abler LL, Eliceiri KW, Huang W, Vezina CM, et al. Characterization of fibrillar collagens and extracellular matrix of glandular benign prostatic hyperplasia nodules. PLoS One. 2014; 9: e109102.

3. Carpentier G, Henault E, Protein Array Analyzer for ImageJ. Proceedings of the ImageJ User and Developer Conference, Centre de Recherche Public Henri Tudor, ed. (ISBN 2-919941- 11-9). 2010; 238-240.

4. Francí C, Takkunen M, Dave N, Alameda F, Gómez S, Rodríguez R, et al. Expression of Snail protein in tumor-stroma interface. Oncogene. 2006; 25: 5134–5144.

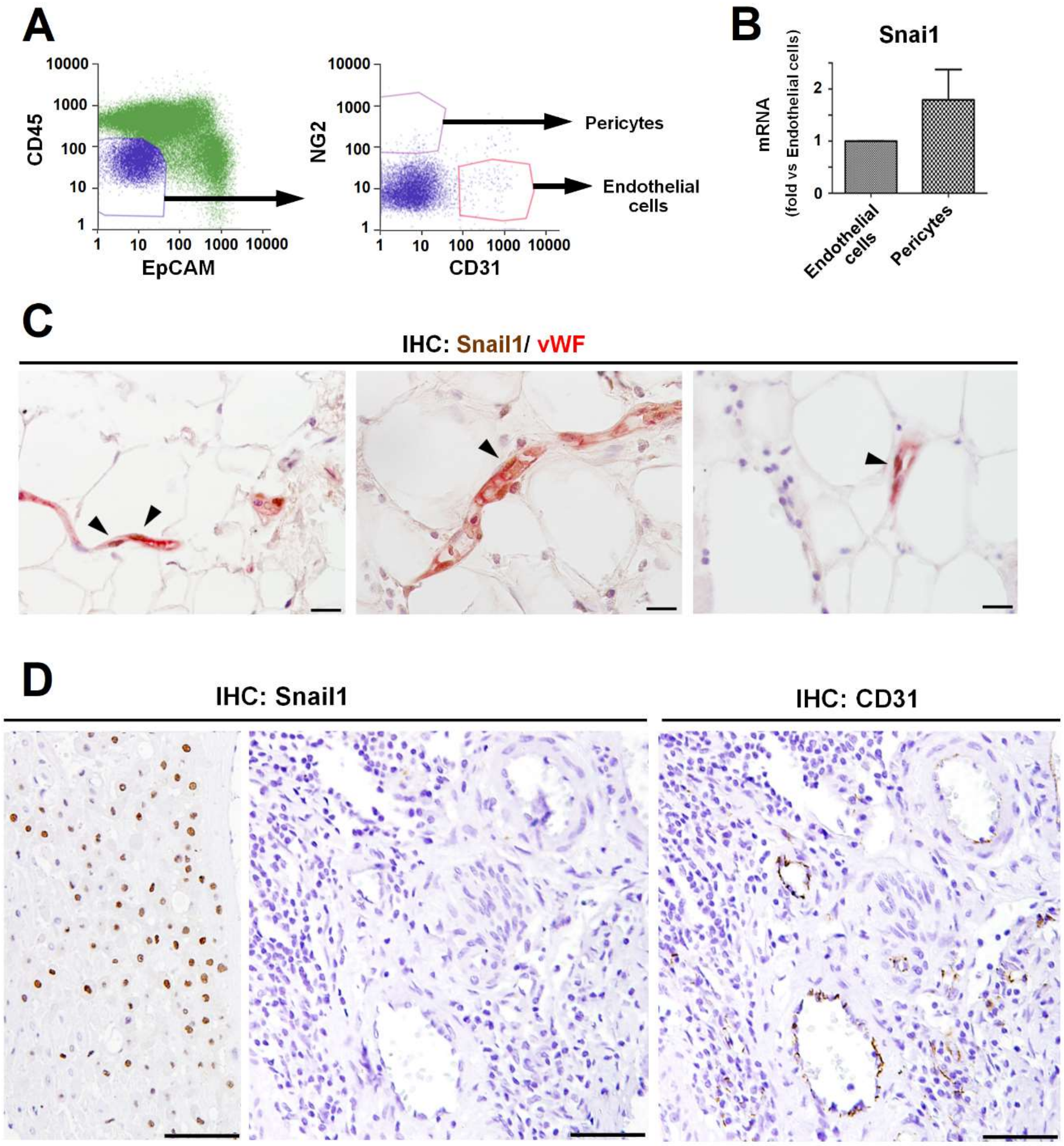

Figure S1. Snail1 is expressed by tumoral endothelial cells and pericytes and not in not-activated endothelium. A, B, Endothelial cells and pericytes were obtained from breast tumors generated in MMTV-PyMT mice. The FACS gating strategy for the purification of these cells is presented in A and the relative levels of *Snai1* mRNA, as determined by qRT-PCR, in B. The figure shows mean ± SD of two independent experiments. **C,** Representative images of Snail1 (in brown) and von Willebrand factor (vWF) (in red) double immunohistochemistry in blood vessels of human colorectal carcinomas. Arrowheads point to Snail1-positive cells. Scale bar: 20 µm. **D,** Immunohistochemical analysis of Snail1 or CD31 expression in non-tumorigenic colon

(middle and right panel). Placenta was used as a positive control for Snail1 expression (left). Scale bars correspond to 50 μm.

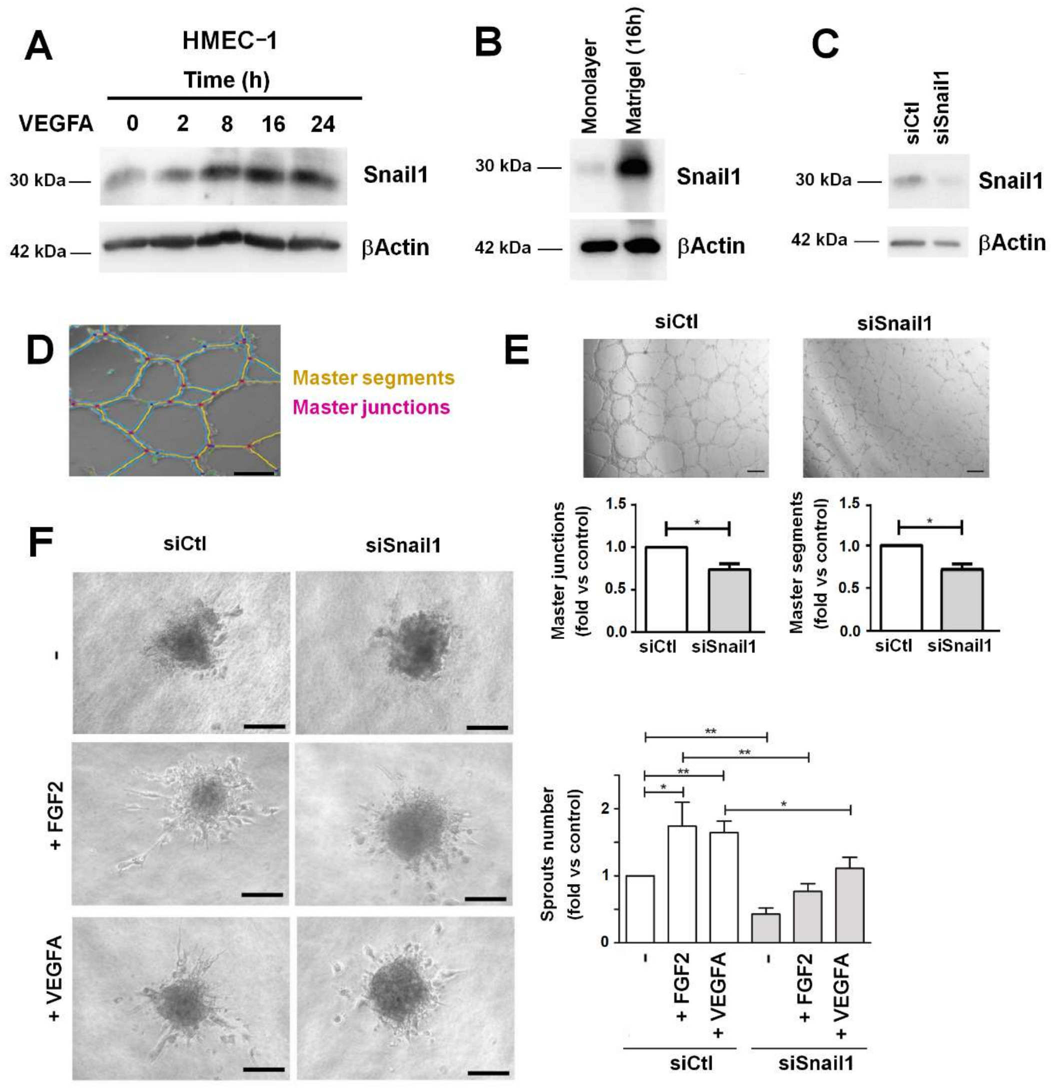

Figure S2. Snail1 depletion compromises angiogenesis and invasion of HMEC-1 cells. A, Western blots analysis of HMEC-1 cells grown in monolayer and treated for the indicated times with VEGFA (80 ng/ml). **B, C,** Snail1 Western blot analysis of total extracts of HMEC-1 grown in monolayer or over Matrigel for 16 h (C) or transfected with siCtl or siSnail1 (D). **D**, Example of the honeycomb panel structure created by HMEC-1 seeded over Matrigel for 16 h and showing the master segments (in yellow) and the master junctions (in pink). Scale bar: 200 μm. **E,** Representative images of the honeycomb panel structures created by of HMEC-1 cells transfected with siCtl or siSnail1 and seeded over Matrigel during 16 h. The quantification of master junctions and master segments (fold vs control) is shown below. **F,** Representative images of siCtl or siSnail1 transfected HMEC-1 spheroids embedded in Collagen type 1 and cultured with DMEM plus VEGFA or FGF2 when indicated. Scale bar: 40 μm. The

quantification of Collagen type I induced sprouts (fold vs control) is presented at the right. Data represent mean ± SEM of at least three independent experiments. * $P < 0.05$; ** $P < 0.01$.

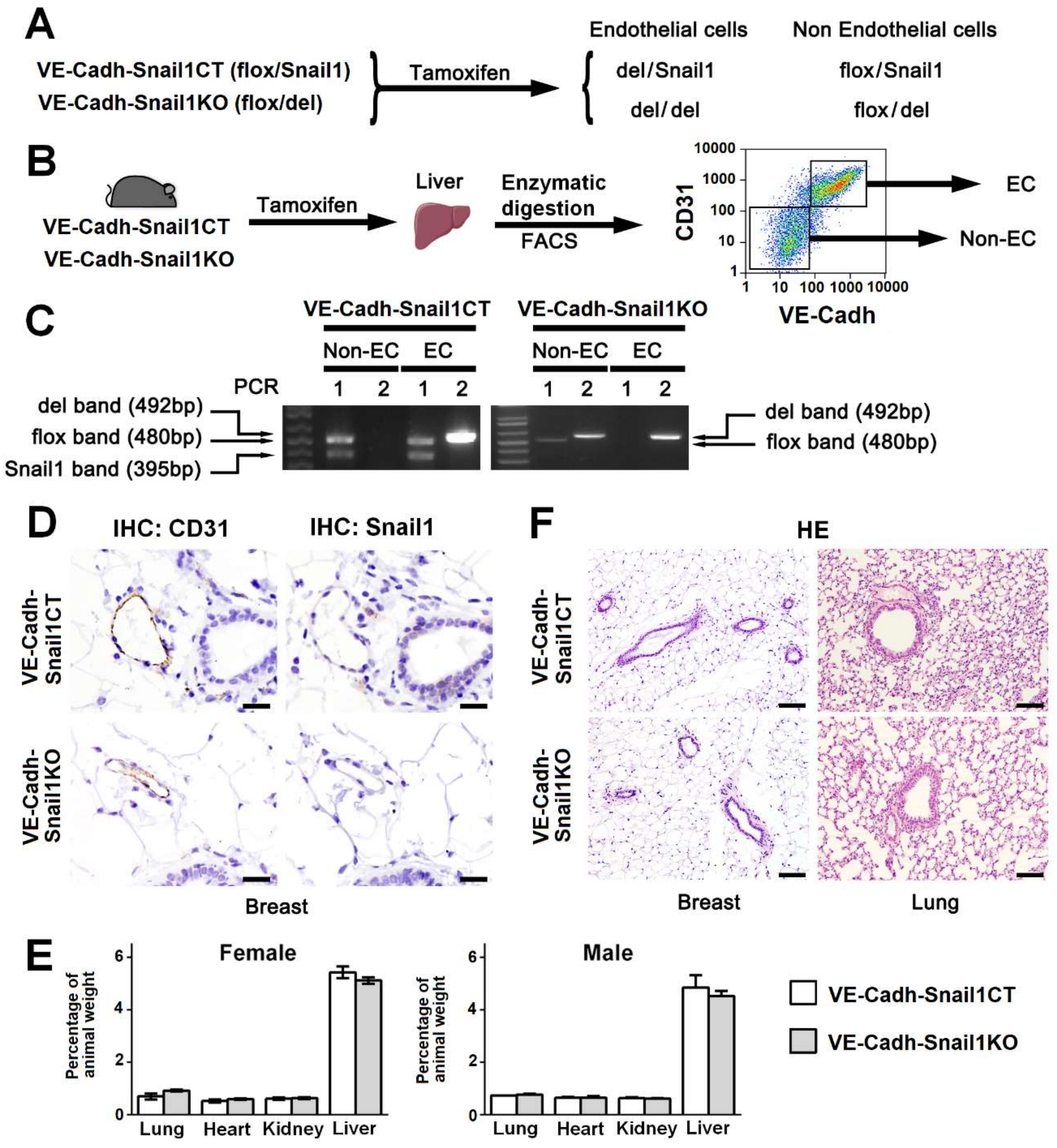

Figure S3. Generation of a murine line with endothelium-specific Snail1 depletion. A, Scheme of genomic Snail1 loxP cassette recombination after tamoxifen treatment in VE-Cadh^{Snail1CT} and VE-Cadh^{Snail1KO} mice. **B,** FACS gating strategy for endothelial (EC) and non-endothelial cells (Non-EC) isolation from mice liver. **C,** Results of the PCR analysis used for genotyping of VE-Cadh^{Snail1CT} and VE-Cadh^{Snail1KO} mice, and comparing endothelial (EC) and non-endothelial cells (Non-EC). The three different fragments amplified (*Snai1 WT*, *flox* and *del* bands) are shown. **D**, CD31 and Snail1 immunohistochemistry analysis of VE-Cadh^{Snail1CT} and VE-Cadh^{Snail1KO} female mice breasts. Scale bar: 20 μm. **E,** Weight of lung, heart, kidney and liver from adult female and male VE-Cadh^{Snail1CT} and VE-Cadh^{Snail1KO} mice is represented as percentage of the total animal weight three weeks after tamoxifen treatment. **F**,

Hematoxylin and eosin stained images of VE-Cadh^{Snail1CT} and VE-Cadh^{Snail1KO} mice breasts and lungs. Scale bar: 200 μm.

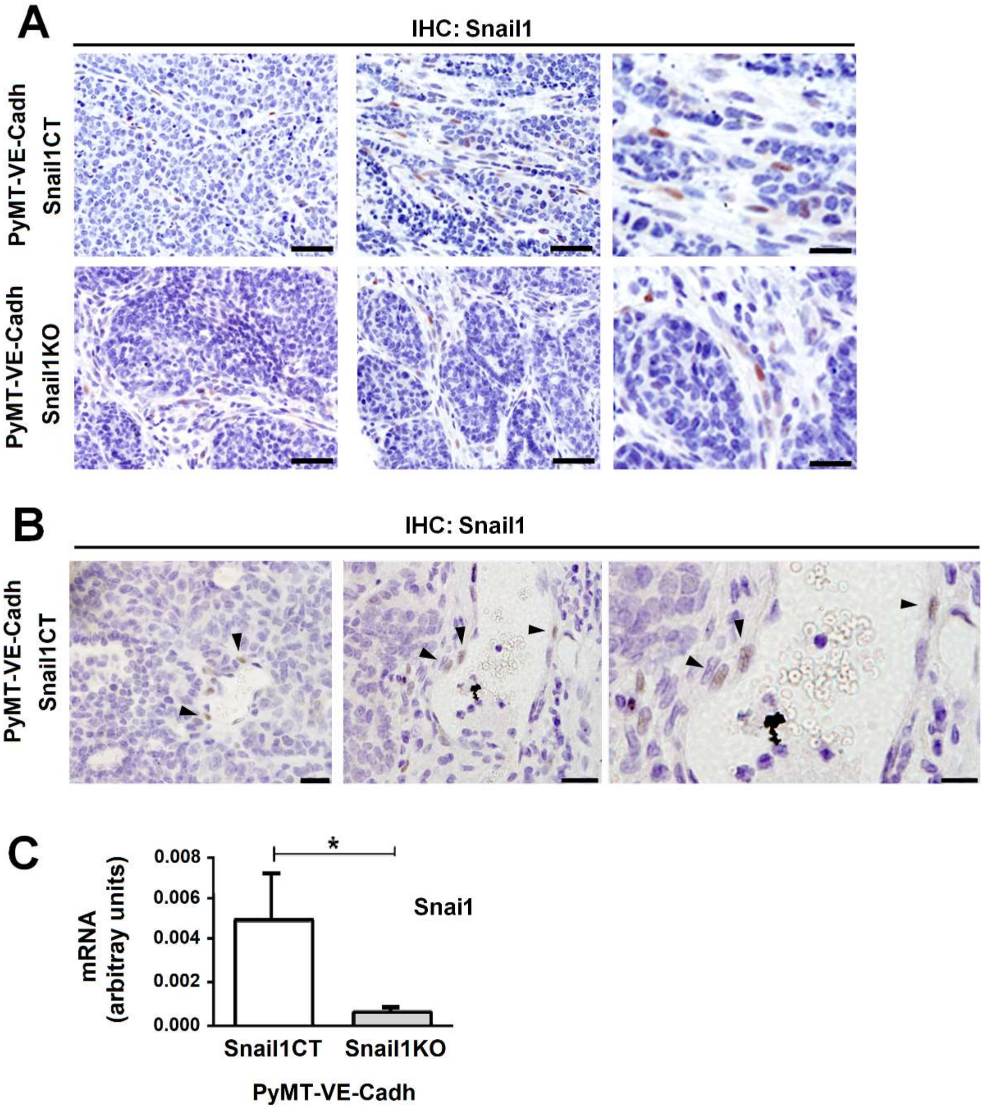

Figure S4. Snail1 expression in MMTV-PyMT tumors. A, B, Representative images of Snail1 staining of PyMT-VE-Cadh^{Snail1CT} and PyMT-VE-Cadh^{Snail1KO} carcinomas showing Snail1 stromal expression in fibroblast-like (A) and endothelial cells (B). Nuclei were counterstained with hematoxylin. The right column presents a higher magnification of the middle column. Black arrowheads point Snail1-positive cells cells. Scale bars: 50 μm, A, left and middle columns; 100 μM, A, right column; 20 μm, B, left and middle columns; 40 μm, right column. **C**, *Snai1* mRNA levels were determined by qRT-PCR in tumor endothelial cells isolated as indicated in Methods from PyMT-VE-Cadh^{Snail1CT} and PyMT-VE-Cadh^{Snail1KO} carcinomas. Data represent mean ± SEM of at least three mice. * $P < 0.05$.

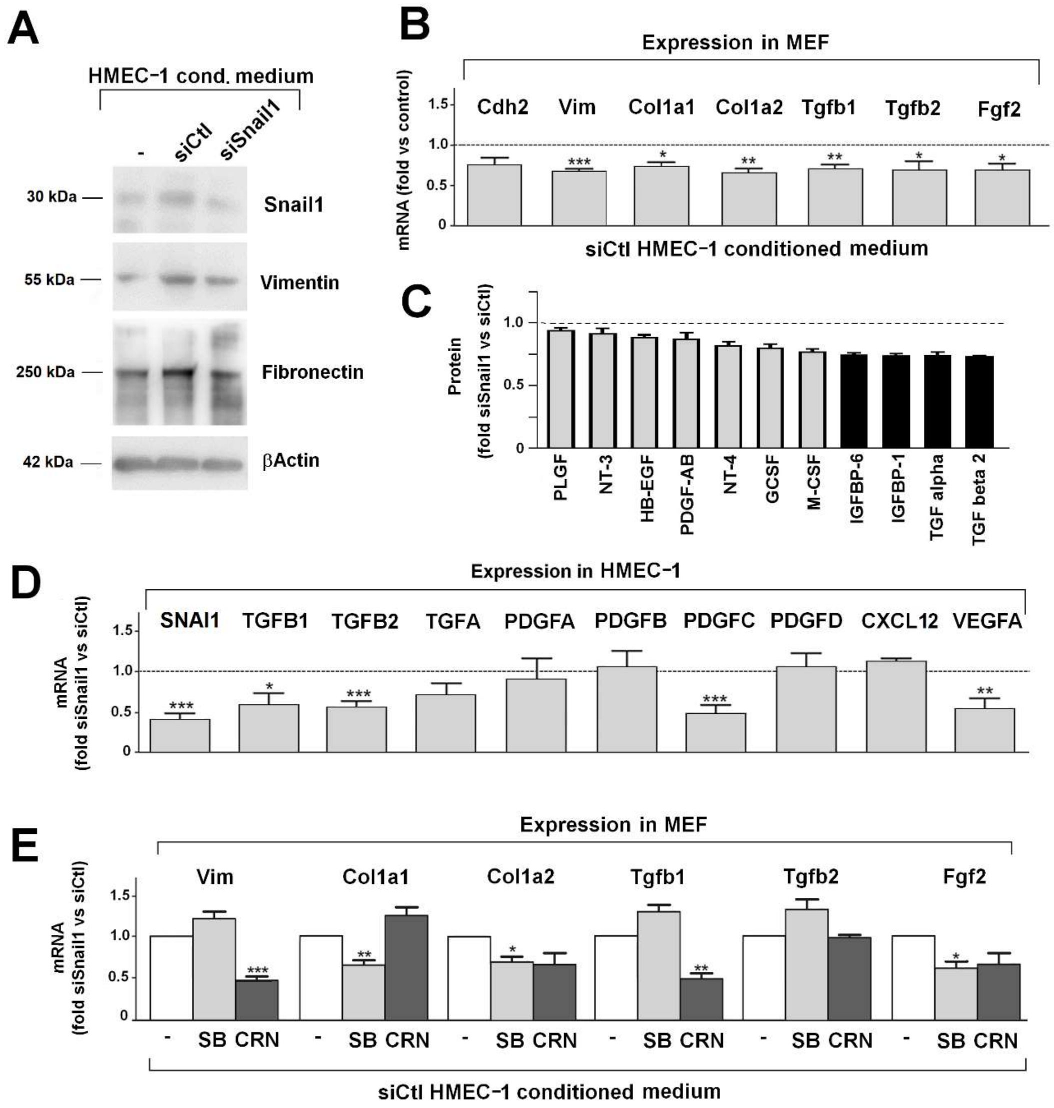

Figure S5. Conditioned medium from HMEC-1 cells stimulates fibroblasts. A-B, MEF were cultured with conditioned medium from siSnail1 or siCtl HMEC-1 for 2 days and the expression of the indicated proteins (A) or mRNAs (B) were carried out by Western blot or qRT-PCR. In B, values with respect to the corresponding control are shown. **C**, siSnail1 or siCtl HMEC-1 were maintained in regular culture medium without FBS for 48 h. The analysis of the conditioned media was performed using Human Growth Factor Antibody Array C1 (RayBiotech C-Series, AAH-GF-1-2) according to manufacturer's instructions. After developing the membranes, the signal was quantified by Protein Array Analyzer for ImageJ [3]. The figure shows mean ± SEM of the duplicated signal for the indicated factors. Expression of proteins down-regulated in siSnail1 versus siCtl HMEC-1 cells are presented; black bars correspond to proteins

that were decreased more than 25% in the conditioned medium. **D**, qRT-PCR analysis of the expression of the indicated genes in siSnail1 HMEC-1 cells with respect to the control (siCtl). Data represent mean ± SEM of at least three independent experiments. **E**, MEF were cultured with conditioned medium from siCtl HMEC-1 for 2 days in the presence of the inhibitors of TGFβ receptor SB505125 (SB) (5 μM) or PDGF receptor Crenolanib (CRN) (1 μM). The expression of the indicated mRNAs was assessed by qRT-PCR. * $P < 0.05$; ** $P < 0.01$; *** $P < 0.001$.

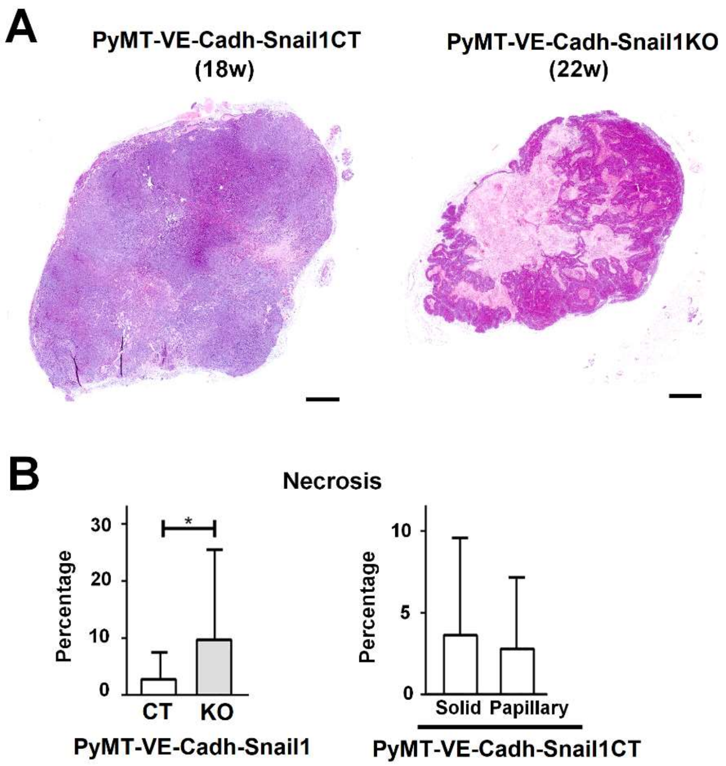

Figure S6. Endothelial Snail1 depletion increases tumor necrosis. (**A**), PyMT-VE-Cadh^{Snail1CT} and PyMT-VE-Cadh^{Snail1KO} mice tumors were collected at 18-week or 22-week old mice, respectively and stained with hematoxylin and eosin. Scale bar, 1 mm. (**B**), Slides were scanned and the necrotic area of PyMT-VE-Cadh^{Snail1CT} and PyMT-VE-Cadh^{Snail1KO} tumors was measured by ImageJ as the percentage of necrosis per total tumor area. Data represent mean ± SEM of at least three tumors of six animals per condition. * $P < 0.05$.

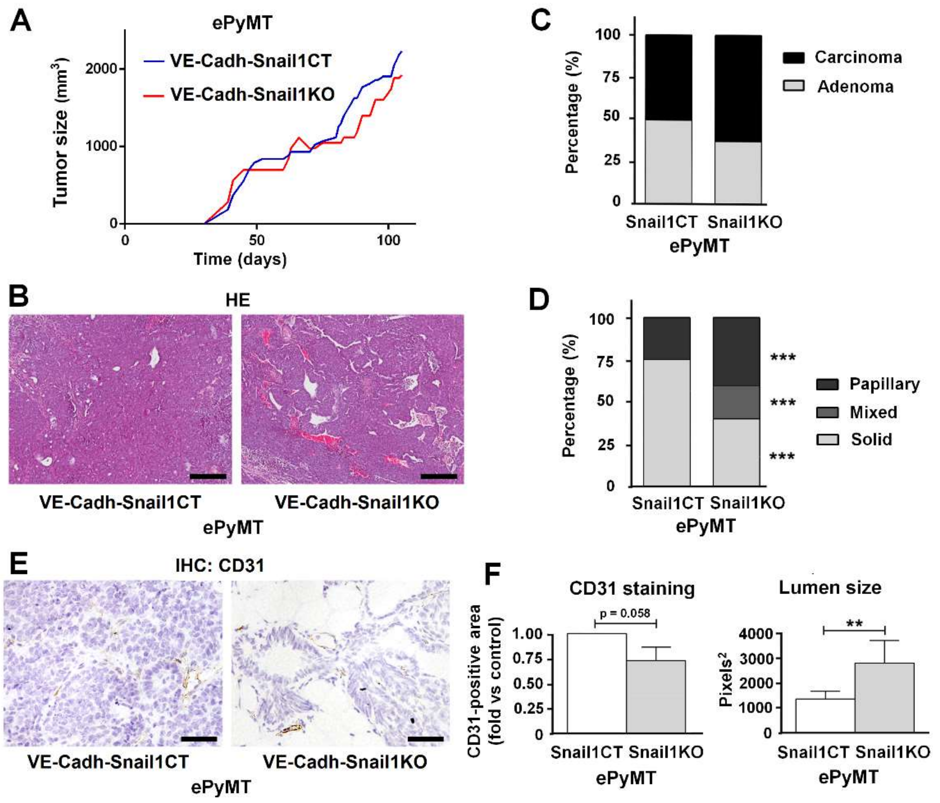

Figure S7. Tumors from grafted ePyMT tumor cells present a different morphology in endothelium Snail1-deficient syngeneic mice. A, Tumor growth of grafted ePyMT tumor cells in syngeneic VE-Cadh^{Snail1CT} and VE-Cadh^{Snail1KO} mice. **B,** Hematoxylin and eosin staining of grafted ePyMT tumors. Scale bar: 250 μm. **C-D,** Quantification of tumor stage (C) and carcinoma morphology (D) of grafted ePyMT tumors in syngeneic VE-Cadh^{Snail1CT} and VE-Cadh^{Snail1KO} mice. **E**, Representative images of the CD31 analysis in these tumors. Scale bar: 50 μm. **F**, Quantification of CD31 staining (left) or vessel lumen size (right) of grafted ePyMT tumors. The graphs represent mean ± SEM. ** $P < 0.01$; *** $P < 0.001$.

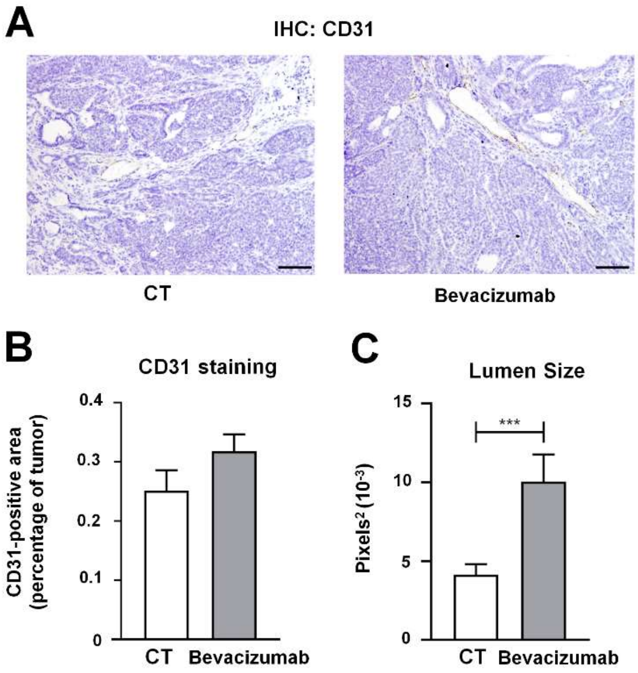

Figure S8. **Bevacizumab-treated MMTV-PyMT mice show tumor vessels with a greater lumen**. **A**, Representative images of the CD31 analysis of control (CT) or Bevacizumab treated tumors obtained at the end-point. Scale bar: 200 μm. Quantification of CD31 staining (**B**) or vessel lumen size (**C**). The graphs represent mean ± SEM. *** $P < 0.001$.

Table S1. Antibodies used in this article

Antibody	Company	Method
βActin	Abcam (ab8227)	WB (1/2000)
CD31	Abcam (ab231436)	IHC (1/200)<br>WB (1/750)
CD31	Biolegend (102515)	FACS (1/200)
CD326 (EpCAM)	Biolegend (118217)	FACS (1/100)
CD45	Pharmigen (557235)	FACS (1/200)
CD45	Abcam (ab208022)	IHC (1/300)
CK14	Abcam (ab181595)	IHC (1/500)
Estrogen Receptor α	Santa Cruz (sc-8005)	IHC (1/50)
Fibronectin1	Abcam (ab2413)	WB (1/1000)
Her2/Neu	BioGenex (MU134-USE)	IHC (1/60)
Ki67	BD (550609)	IHC (1/100)
Lamininα	Sigma (L9393)	IHC (1/100)
p63	Santa Cruz (sc-25268)	IHC (1/40)
PhosphoSmad2 (Ser465-467)	Cell Signalling (3108)	IHC (1/100)
Snail1	Francí et al 2006 [4]	IHC (1/100)
Snail1	Cell Signaling (3879)	WB (1/2000)<br>IF (1/150)<br>IHC (1/100)
VE-Cadherin	Pharmigen (562242)	FACS (1/50)
VE-Cadherin	Santa Cruz (sc-52751)	WB (1/200)
Vimentin	BD (550513)	IHC (1/200)<br>WB (1/500)
Factor VIII Related Antigen/<br>von Willebrand Factor	Thermo (RB-281-A1)	IHQ (1/100)

WB: Western Blot; FACS: Fluorescence-Activated Cell Sorter; IF: Immunofluorecence; IHC: Immunohistochemistry.

Table S2. Primers used in qRT-PCR analysis

Gene	Forward	Reverse
**Human**		
CXCL12	5’-CAGTCAGCCTGAGCTACCGA-3’	5’-GCCGTGCAACAATCTGAAGG-3’
HPRT	5’-GGCCAGACTTTGTTGGATTTG-3’	5’-TGCGCTCATCTTAGGCTTTGT-3’
PDGFA	5’- GCAAGACCAGGACGGTCATTT-3’	5’-GGCACTTGACACTGCTCGT-3’
PDGFB	5’-CTCGATCCGCTCCTTTGATGA-3’	5’-CGTTGGTGCGGTCTATGAG-3’
PDGFC	5’-TTCTTGGCAAGGCTTTTGTT-3’	5’-TGCTTGGGACACATTGACAT-3’
PDGFD	5’-GTGGAGGAAATTGTGGCTGT-3’	5’-CGTTCATGGTGATCCAACTG-3’
SNAI1	5’-GTGCCTCGACCACTATGCC-3’	5’-GCTGCTGGAAGGTAAACTCTGG-3’
TGFA	5’-CGCCCTGTTCGCTCTGGGTATTG-3’	5’-CGGTGATGGCCTGCTTCTTCTGG-3’
TGFB1	5’-CGTCTGCTGAGGCTCAAGTTAAA-3’	5’-CCGGTAGTGAACCCGTTGAT-3’
TGFB2	5’-TCCCAGGTTCCTGTCTTTATG-3’	5’-GATGCCATCCCGCCCACTTTC-3’
VEGFA	5’-CCAACTGAGGAGTCCAACAT-3’	5’-TTTCTTGCGCTTTCGTTTTT-3’
**Murine**		
Cdh2	5’-GCAGGTACCATGCTGACCAC-3’	5’-CCAGTTGGCAGGATCAGAC-3’
Col1a1	5’-CGCCATCAAGGTCTACTGC-3’	5’-ACGGGAATCCATCGGTCA-3’
Col1a2	5’-CTGGTCCTGTTGGAAGTCGT-3’	5’-CAGATGCACCTGTTTCTCCA-3’
Fgf2	5’-GACCCACACGTCAAACTACAA-3’	5’-GTAACACACTTAGAAGCCAGCAG-3’
Hprt	5’-GGCCAGACTTTGTTGGATTTG-3’	5’-TGCGCTCATCTTAGGCTTTGT-3’
Snai1	5´-GCGCCCGTCGTCCTTCTCGTC-3´	5´-CTTCCGCGACTGGGGGTCCT-3´
Tgfb1	5’-CTGCAAGACCATCGACATGG-3’	5’-GTTCCACATGTTGCTCCACA-3’
Tgfb2	5’-CTACAGACTGGAGTCACAAC-3’	5’-CATATTGGAAAGCTGTTCGATC-3’
Vim	5’-GGCTGCGAGAGAAATTGC-3’	5’-TCTCTTCATCGTGCAGTTTCTTC-3’
